# Brain Structural Correlates of EEG Network Hyperexcitability, Symptom Severity, Attention, and Memory in Borderline Personality Disorder

**DOI:** 10.3390/brainsci15060592

**Published:** 2025-05-31

**Authors:** Andrea Schlump, Bernd Feige, Swantje Matthies, Katharina von Zedtwitz, Isabelle Matteit, Thomas Lange, Kathrin Nickel, Katharina Domschke, Marco Reisert, Alexander Rau, Markus Heinrichs, Dominique Endres, Ludger Tebartz van Elst, Simon Maier

**Affiliations:** 1 Department of Psychiatry and Psychotherapy, Medical Center-University of Freiburg, Faculty of Medicine, University of Freiburg, 79085 Freiburg, Germanybernd.feige@uniklinik-freiburg.de (B.F.); tebartzvanelst@uniklinik-freiburg.de (L.T.v.E.); 2 Laboratory for Biological Psychology, Clinical Psychology and Psychotherapy, Department of Psychology, University of Freiburg, 79085 Freiburg, Germany; 3 Division of Medical Physics, Department of Diagnostic and Interventional Radiology, Medical Center-University of Freiburg, Faculty of Medicine, University of Freiburg, 79085 Freiburg, Germany; 4 German Center for Mental Health (DZPG), Partner Site Berlin/Potsdam, 10117 Berlin, Germany; 5 Department of Stereotactic and Functional Neurosurgery, Medical Center-University of Freiburg, Faculty of Medicine, University of Freiburg, 79085 Freiburg, Germany; 6 Department of Neuroradiology, Medical Center-University of Freiburg, Faculty of Medicine, University of Freiburg, 79085 Freiburg, Germany

**Keywords:** BPD, cortical thickness, cortical volume, subcortical volume, electroencephalography, IRDA/IRTA

## Abstract

**Introduction**: Previous neuroimaging studies have reported structural brain alterations and local network hyperexcitability in terms of increased slow-wave electroencephalography (EEG) activity in patients with borderline personality disorder (BPD). In particular, intermittent rhythmic delta and theta activity (IRDA/IRTA) has drawn attention in mental health contexts due to its links with metabolic imbalances, neuronal stress, and emotional dysregulation—processes that are highly pertinent to BPD. These functional disturbances may be reflected in corresponding structural brain changes. The current study investigated cortical thickness and subcortical volumes in BPD and examined their associations with IRDA/IRTA events per minute, symptom severity, and neuropsychological measures. **Methods**: Seventy female BPD patients and 36 age-matched female healthy controls (HC) were included (for clinical EEG comparisons even 72 patients were available). IRDA/IRTA rates were assessed using an automatic independent component analyses (ICA) approach. T1-weighted MRI data were obtained using a MAGNETOM Prisma 3T system and analyzed with FreeSurfer (version 7.2) for subcortical structures and CAT12 for cortical thickness and global volume measurements. Psychometric assessments included questionnaires such as Borderline Symptom List (BSL-23) and Inventory of Personality Organization (IPO). Neuropsychological performance was evaluated with the Test for Attentional Performance (TAP), Culture Fair Intelligence Test (CFT-20-R), and Verbal Learning and Memory Test (VLMT). **Results**: Between-group comparisons exhibited no significant increase in IRDA/IRTA rates or structural abnormalities between the BPD and HC group. However, within the BPD group, cortical thickness of the right isthmus of the cingulate gyrus negatively correlated with the IRDA/IRTA difference (after minus before hyperventilation, HV; *p* < 0.001). Furthermore, BPD symptom severity (BSL-23) and IPO scores positively correlated with the thickness of the right rostral anterior cingulate cortex (*p* < 0.001), and IPO scores were associated with the thickness of the right temporal pole (*p* < 0.001). Intrinsic alertness (TAP) significantly correlated with relative cerebellar volume (*p =* 0.01). **Discussion**: While no group-level structural abnormalities were observed, correlations between EEG slowing, BPD symptom severity, and alertness with cortical thickness and/or subcortical volumes suggest a potential role of the anterior cingulate cortex, temporal pole, and cerebellum in emotion regulation and cognitive functioning in BPD. Future research employing multimodal EEG-MRI approaches may provide deeper insights into the neural mechanisms underlying BPD and guide personalized therapeutic strategies.

## 1. Introduction

Borderline personality disorder (BPD) is a severe mental health condition, affecting approximately 1–3% of the general population [1,2,3,4]. It is characterized by pervasive patterns of emotional instability, impulsivity, inconsistent self-image, along with disturbed interpersonal functioning and debilitating psychosocial implications [5,6,7]. Due to deficits in emotion regulation, many individuals exhibit maladaptive coping mechanisms such as repetitive self-harm, suicidal behavior, and episodic dissociative phenomena in response to states of inner tension and acute stress. The heterogeneous manifestations of BPD symptoms require a systematic comprehension of the underlying pathophysiology to advance personalized treatment approaches in clinical practice [8,9]. In recent years, the investigation of potential biomarkers linked to BPD symptomatology has become increasingly important, with structural neuroimaging playing a crucial role [10]. In magnetic resonance imaging (MRI), voxel- and surface-based morphometry (SBM) allow for an objective assessment of structural brain parameters such as cortical thickness and volume. Numerous studies have identified morphometric abnormalities in BPD, with meta-analyses reporting gray matter (GM) alterations in brain regions that are part of the default mode network including the precuneus, anterior, as well as posterior cingulate cortices and fronto-limbic regions involving the orbitofrontal gyrus, amygdala and hippocampus [11,12,13,14,15,16], temporal gyri, and supplementary motor area [17].

However, several studies have reported divergent findings. For example, Bruehl et al. [18] and Vai et al. [19] found no significant volume reductions of the amygdala and hippocampus in BPD. Similarly, inconsistencies exist regarding prefrontal cortical thickness, with some studies reporting increases [18], while others indicate cortical thinning in the same region [20,21]. These discrepancies may arise from differences in sample characteristics, comorbid conditions, methodology, and symptom heterogeneity across patients with BPD. Yet, structural brain abnormalities in BPD are predominantly observed in regions involved in emotion regulation, impulse control, and social cognition.

Neuropsychological deficits in BPD are also well-documented, with some studies exploring their relationship with symptom severity and cortical thickness [19,22]. Vai and colleagues [19], for example, demonstrated that impairments in psychomotor speed and coordination were positively associated with cortical thickness in the postcentral gyrus.

Complementing neuroimaging, electroencephalography (EEG) has been discussed as a diagnostic biomarker for BPD subgroups. A link between epilepsy and BPD has been discussed for some time [23]. Studies have identified a higher prevalence of diffuse EEG abnormalities in BPD patients, including pathological slow-wave activity [24,25,26]. Particularly, intermittent rhythmic delta and theta activity (IRDA/IRTA) patterns on the EEG, first documented by Cobb [27], have been associated with local network hyperexcitability. This hyperexcitability may result from focal structural lesions, epilepsy or epileptiform discharges, and metabolic or inflammatory disturbances [28,29,30], and could reflect adaptive processes in neuronal networks [31,32,33,34,35]. A case study of a patient with BPD found that valproate treatment was associated with a decrease in IRDA/IRTA frequency and a concurrent reduction in clinical symptoms, including dissociation and self-harm [34]. Building upon that finding, a retrospective study compared the prevalence of EEG anomalies in 96 patients with BPD and 76 healthy controls (HC). The study demonstrated significantly higher mean IRDA/IRTA rates in the BPD group (14.6%) compared to HC (3.9%) [35]. Taken together, integrating findings from structural neuroimaging, EEG, and neuropsychological measures may provide a more comprehensive understanding of BPD symptomatology.

The rationale of this study was to compare cortical thickness, as well as subcortical and cortical volumes in women with and without BPD, and to explore potential associations of these brain structural measures and IRDA/IRTA rates per minute (based on our earlier findings). Such multimodal approaches represent a significant advance in the understanding of the neurobiology of BPD. We hypothesized that women with BPD exhibit higher IRDA/IRTA rates and morphometric abnormalities—specifically reductions in GM and white matter (WM) volume, and that these changes are associated with higher IRDA/IRTA rates per minute. Furthermore, we examined whether these structural alterations were associated with psychometric and neuropsychological measures.

## 2. Methods

This study was part of a larger research project investigating the relationship between slow-wave EEG activity and MRI-derived measures. Ethical approval was obtained from the University Medical Centre Freiburg ethics committee (application no. EK-Freiburg: 209/18), and all participants provided written informed consent.

### 2.1. Patient Assessment

Women aged ≥ 18 years with BPD were recruited from the specialized inpatient unit for BPD at the Department of Psychiatry and Psychotherapy, University Hospital Freiburg. Experienced clinicians established the BPD diagnosis according to the ICD-10 criteria during an observation period of multiple weeks involving inpatient dialectical behavior therapy (DBT). Additionally, all patients met diagnostic criteria for BPD on the Structured Clinical Interview for DSM-IV Axis II Disorders (SCID-II; ≥ 5 of 9 criteria). Exclusion criteria included lifetime diagnoses of schizophrenia, bipolar disorder, or acute psychotic symptoms, substance dependence except for episodic abuse or lack of abstinence for ≥ 6 months prior to screening. Antiepileptic drugs prescribed for epilepsy, seizures, pregnancy, lactation, relevant physical illness interfering with study assessments, brain injury or neurological diseases (e.g., hydrocephalus, space-occupying processes, traumatic brain injury, encephalitis, meningitis, and seizures/epilepsy), inability to provide informed consent, and any MR contraindications (e.g., pacemaker, and intrauterine device) also led to exclusion.

### 2.2. Healthy Control Group Assessment

The HC group was recruited through public announcements. Adult female HC aged ≥ 18 years were included. Lifetime diagnosis of an axis I or II disorder according to DSM-IV (assessed and confirmed using SCID-I screening and SCID-II screening for BPD) led to study exclusion. Additional exclusion criteria included: recent substance use within the last six months (episodic cannabis use did not result in study exclusion), history of psychopharmacological medication, pregnancy or lactation, significant physical illness that could affect study outcomes, history of major brain injury or neurological diseases (e.g., traumatic brain injury, encephalitis, meningitis, seizures/epilepsy, hydrocephalus, systematic autoimmune diseases with possible brain involvement, and space-occupying processes) inability to provide informed consent, and MRI contraindications (pacemakers, intrauterine device, etc.).

### 2.3. Sociodemographic, Psychometric, and Neuropsychological Testing

Sociodemographic data including education, employment status, handedness, and comorbidities were collected using a self-report questionnaire. An overview of the psychometric and neuropsychological test battery is provided in Supplementary Table S1. Missing test data did not lead to study exclusion if participants met the above-mentioned eligibility criteria and, in case of patients, had a clinically confirmed BPD diagnosis.

### 2.4. Study Cohort

**Figure 1:**
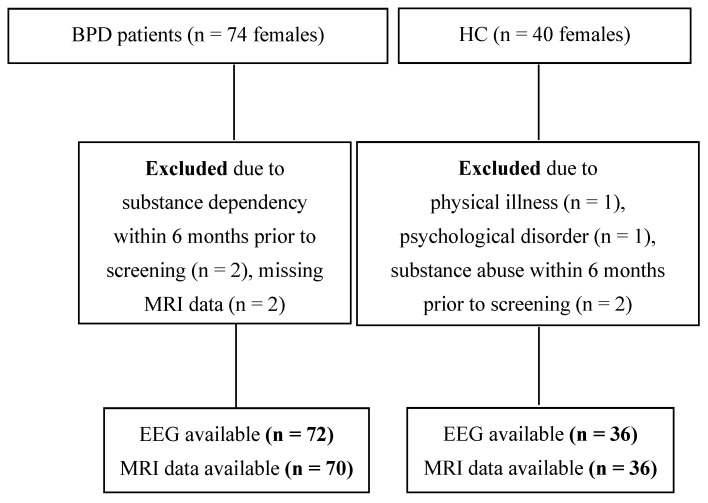
Participant recruitment flow chart. Abbreviations: BPD, borderline personality disorder; EEG, electroencephalography; HC, healthy controls; MRI, magnetic resonance imaging; n, sample size.

The recruitment flow-chart is depicted in Figure 1. A total of seventy-four patients with BPD were enrolled, of whom 70, along with 36 HCs, were included in the EEG/MRI analyses. For EEG analyses, data from 72 patients and 36 age-matched HCs were available.

### 2.5. EEG IRDA/IRTA Analysis

Clinical EEG data were recorded with a Nihon Kohden Neurofax EEG-1200 system (Nihon Kohden Corp., Tokyo, Japan) using the 10–20 montage (21 Ag-AgCl sintered bridge electrodes). Electrode impedances were kept below 5 kOhm. The standardized recording session lasted 11 min with eyes open/close maneuvers and 3 min hyperventilation (HV). IRDA/IRTA was identified using an in-house software (available at https://github.com/berndf/avg_q (accessed on 27 May 2025)). IRDA/IRTA rates were quantified as the number of events per minute, both before and during HV, as well as the difference in event rates during HV compared to before HV (IRDA/IRTA difference). This methodology has been previously described in detail by Endres et al. [32]. The IRDA/IRTA amplitude threshold was set to >1 µV.

### 2.6. MRI Acquisition and Analysis

All MRI measurements were conducted on a 3 Tesla MAGNETOM Prisma 3T system (Siemens Healthineers^®^, Erlangen, Germany) equipped with a 64-channel head and neck coil for final reception. Structural MRI data were acquired using a T1-weighted magnetization-prepared rapid gradient echo (MPRAGE) sequence with the following parameters: repetition time (TR) = 2000 ms, echo time (TE) = 4.11 ms, field of view (FOV) = 256 × 256 × 160 mm^3^, and isotropic voxel size = 1 × 1 × 1 mm^3^.

Global brain volumes and cortical thickness were analyzed using the Computational Anatomy Toolbox (CAT12; https://dbm.neuro.uni-jena.de/cat/ (accessed on 27 May 2025)) implemented in SPM12, applying default parameters for bias correction, tissue segmentation, and spatial normalization. Subcortical structures were examined with FreeSurfer version 7.2 [36] using the recon-all pipeline [37] and its default steps (e.g., intensity normalization, skull stripping, and segmentation).

To explore voxel-based morphometric (VBM) differences in GM volume of BPD patients, voxel-based group comparisons using probability maps derived from [38] have been performed. Age was included as a nuisance covariate in the analysis, which was conducted with the Statistical Parametric Mapping Voxel-Based Morphometry (SPM-VBM) 8 toolbox and threshold-free cluster enhancement (TFCE; https://github.com/markallenthornton/MatlabTFCE (accessed on 27 May 2025)). Correction for multiple comparisons across voxels was applied using the family-wise error (FWE) method.

### 2.7. Statistical Analyses

All statistical analyses were conducted using R software version 3.6.0 (R Foundation for Statistical Computing Platform, Vienna, Austria). Group differences in categorical variables (e.g., sex) were performed using Fisher’s exact test. The Shapiro–Wilk test was applied to evaluate the normality of continuous variables. For normally distributed data, independent samples *t*-tests were used; if the normality assumption was violated, robust Yuen’s tests (yuenbt) [39] were performed. All brain structural measures were adjusted for age and the image quality rating derived from CAT12 (IQR) using the predict function in linear models. Specifically, each measure was regressed on these covariates using a linear model in R, and the residuals (i.e., the variance not explained by the covariates) were extracted via the predict function for subsequent statistical analyses. Although the initial goal was to recruit equal numbers of BPD patients with clinically detectable IRDA/IRTA (N = 36) and without IRDA/IRTA (N = 36), assembling the IRDA/IRTA subgroup proved infeasible. Consequently, a correlational approach was adopted. Within the BPD group, robust correlation analyses were performed using the pbcor function of the WRS2 R package [40]. Effect sizes were calculated in R using Cohen’s d for robust statistics [40,41]. All *p*-values were corrected for multiple testing using the Benjamini–Hochberg procedure [42], with statistical significance set at *p* < 0.05.

## 3. Results

### 3.1. Study Cohort

Both the BPD and HC groups consisted of female participants, with no significant difference in age (BPD: 30.14 ± 9.5, HC: 27.08 ± 7.4; *p* = 0.71). However, patients with BPD had significantly lower educational attainment (*p* < 0.001) and were more frequently unemployed or retired (*p* < 0.001). Sociodemographic data are summarized in Supplementary Table S2.

Psychiatric comorbidities were reported in 97% of the BPD group but none of the HC (see Table 1). While none of the HC participants were taking psychopharmacological medication, 95.7% of BPD participants were (see Table 2). Substance abuse within the past six months was reported by 10% (n = seven) of the BPD patients, and none of the HC. BPD patients differed significantly in various psychometric and neuropsychological measures from the HC group. Patients with BPD exhibited significantly higher scores on the Borderline Symptom List-23 (42.71 ± 19.8; *p* < 0.001), the BSL-Supplement (3.28 ± 8.7; *p =* 0.018), the Difficulties in Emotion Regulation Scale (DERS, 54.34 ± 24.3; *p* < 0.001), and higher dissociation scores measured by the Dissociative Experience Scale (FDS-20, 16.59 ± 14.3; *p* < 0.001). Psychometric and neuropsychological measures are presented in Table 3.

### 3.2. Group Differences in Electroencephalography Findings

**Figure 2:**
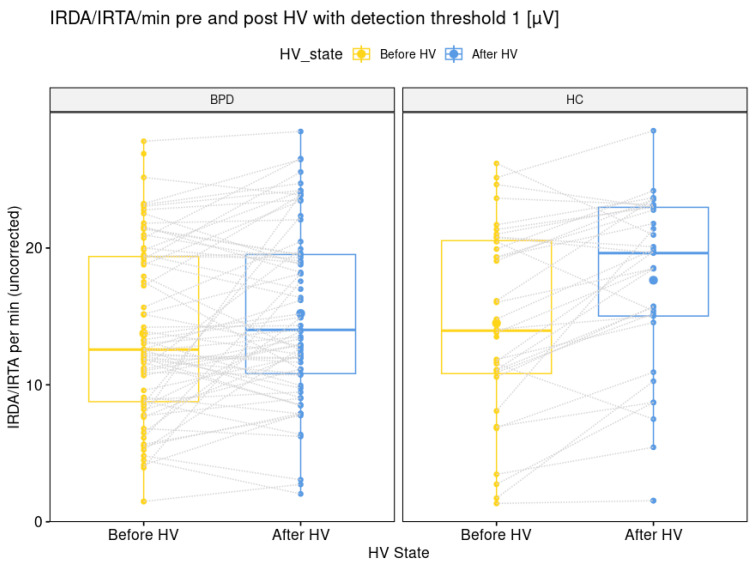
Graphical illustration of intermittent rhythmic delta and theta activity per minute with a detection threshold of one microvolt before and after hyperventilation for the borderline personality disorder patient and healthy control group. Abbreviations: BPD, borderline personality disorder; HC, healthy controls; HV, hyperventilation; IRDA/IRTA, intermittent rhythmic delta and theta activity; µV, microvolt.

The comparison of IRDA/IRTA rates before HV (14 + 7 in BPD versus 15 + 7 in HC; *p* = 0.564) was not significantly different between the BPD and HC group. The IRDA/IRTA difference (1.5 ± 4.5 in BPD versus 2.9 ± 3.9 in HC; *p* = 0.026) showed a significant difference between patients (N = 70) and HC (N = 36). These findings remained stable between the larger BPD group of 72 patients (30.0 + 9.5 years) with available EEG data and the IRDA/IRTA rates of 14 ± 6 before HV (*p* = 0.547) and IRDA/IRTA difference of 1.5 ± 4.5 and the same HC group (*p =* 0.026; see Figure 2).

### 3.3. Group Differences in Structural Magnetic Resonance Imaging Findings

#### 3.3.1. Cortical Thickness

**Figure 3:**
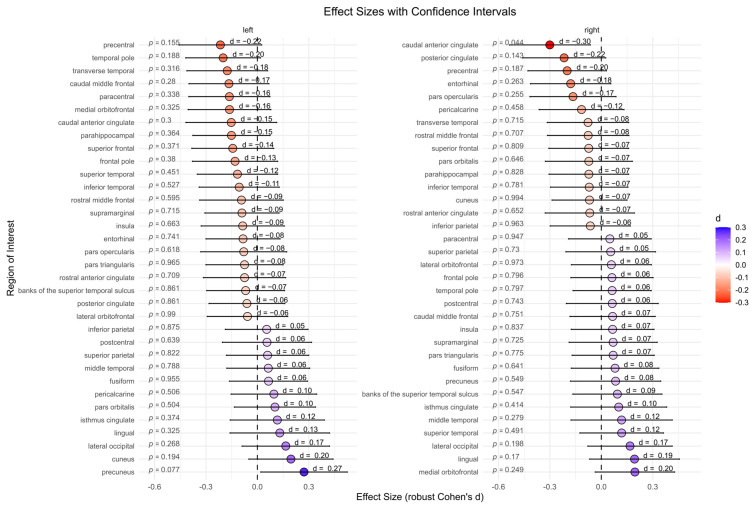
Cortical thickness between the borderline personality disorder patient and healthy control group (not corrected for multiple testing). Abbreviations: d, Cohen’s d.

The BPD patient group showed significantly lower cortical thickness in the right caudal anterior cingulate compared to the HC group (*p =* 0.044, d = −0.30). However, after correcting for multiple comparisons, this effect was no longer significant (*p =* 0.975). No significant differences were found for other subcortical regions (Figure 3).

#### 3.3.2. Global Volumes

**Figure 4:**
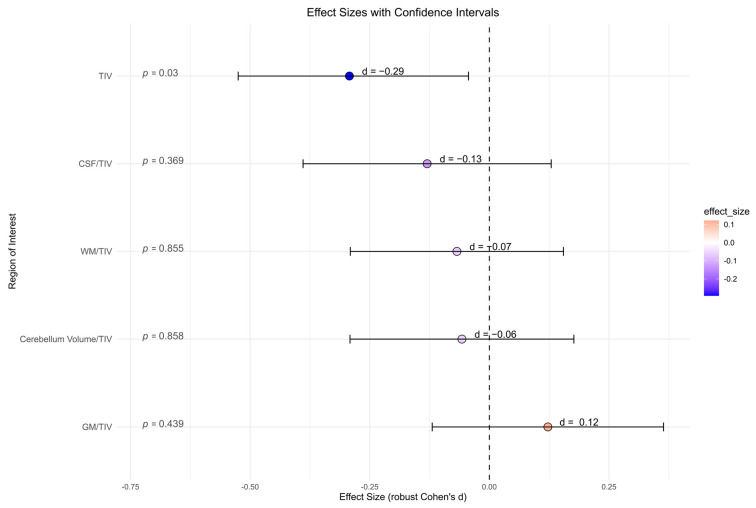
Global volume measures (cerebellum global, CSF, gray matter, white matter) in cubic millimeters from Computational Anatomy Toolbox-12 analyses of the borderline personality disorder patient and healthy control group (not corrected for multiple testing). Abbreviations: CSF, cerebrospinal fluid; d, Cohen’s d; GM, gray matter; TIV, total intracranial volume; WM, white matter.

There were no significant differences between the BPD patient and the HC group for all global volume measures (cerebellum global, CSF, GM, and WM) and its ratio with total intracranial volume, accordingly (Figure 4). BPD patients had a significantly smaller total intracranial volume in contrast to HC (*p =* 0.030, *d* = −0.29). However, after correcting for multiple comparisons, this effect was no longer present (*p =* 0.142).

#### 3.3.3. Subcortical Volumes

**Figure 5:**
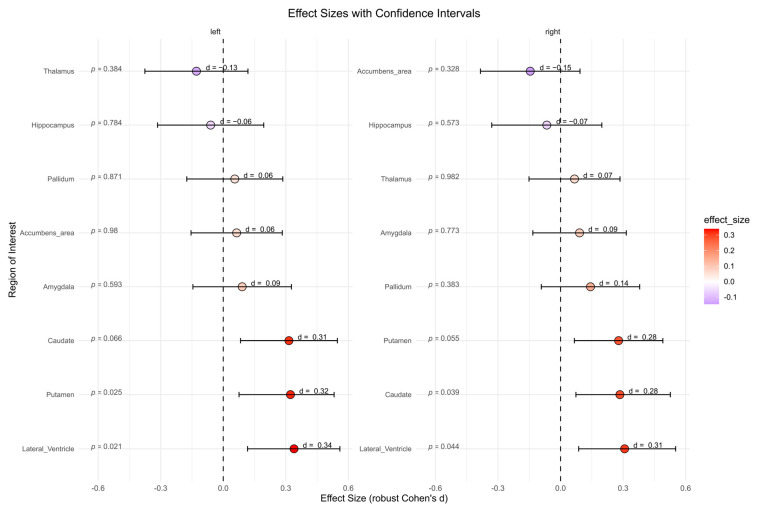
Subcortical volumes in cubic millimeters between the borderline personality disorder patient and healthy control group (not corrected for multiple testing). Abbreviations: d, Cohen’s d.

The BPD patient group showed significantly higher subcortical volumes in left lateral ventricle (*p =* 0.021, *d* = 0.34), right lateral ventricle (*p =* 0.044, *d* = 0.31), left putamen (*p =* 0.025, *d* = 0.32), and right caudate (*p =* 0.039, *d* = 0.28) compared to the HC group (Figure 5). However, after correcting for multiple comparisons, these effects were no longer significant. No significant differences were found for other subcortical regions.

#### 3.3.4. Voxel-Based Group Comparisons

**Figure 6:**
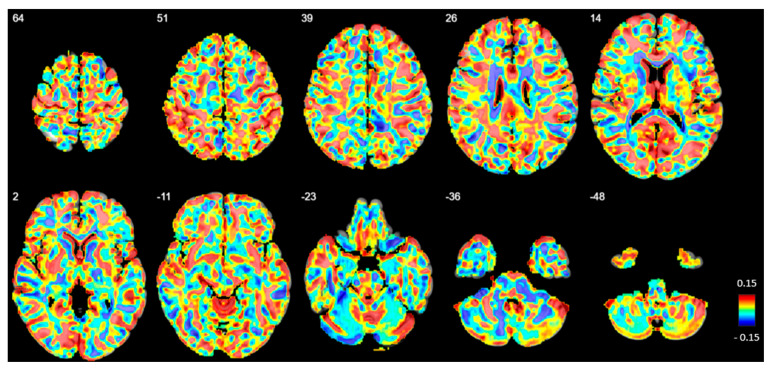
Beta map displaying voxel-based volumetric gray matter differences between patients with borderline personality disorder (BPD) and healthy controls (HC). Positive beta values indicate regions where gray matter volume is reduced in BPD compared to HC. The numbers in the upper left corner represent z-coordinates in MNI space. However, no significant volume differences were observed after family-wise error correction.

No significant differences in GM volume or density were observed between the BPD and HC groups at any voxel (Figure 6).

### 3.4. Correlations with Electroencephalography Findings

#### 3.4.1. Cortical Thickness

**Figure 7:**
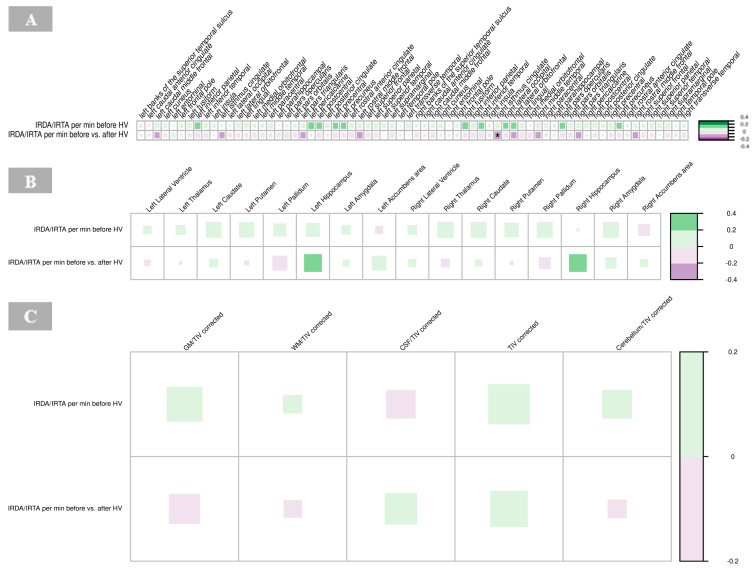
Correlations of IRDA/IRTA per minute with cortical thickness (**A**), subcortical volumes (**B**), and global volumes (gray matter, white matter, cerebrospinal fluid, total intracranial volume, cerebellum) corrected for TIV (**C**) in borderline personality disorder. The color of the boxes in the graph represents the direction of the correlation, with green indicating a positive correlation and purple indicating a negative correlation. The size of each box reflects the strength of the correlation, with larger boxes corresponding to stronger correlations. Significance is indicated by asterisks as follows: * *p* < 0.05; ** *p* < 0.01; *** *p* < 0.001. Abbreviations: CSF, cerebrospinal fluid; GM, gray matter; HV, hyperventilation; IRDA/IRTA, intermittent rhythmic delta/theta activity; TIV, total intracranial volume; WM, white matter.

A significant negative correlation was found between the cortical thickness of the right isthmus of the cingulate gyrus and IRDA/IRTA difference (*p =* 0.001, Supplementary Table S3). No other significant correlations were observed between cortical regions and IRDA/IRTA rates in the BPD group (Figure 7A).

#### 3.4.2. Subcortical Volumes

There were no significant correlations between IRDA/IRTA rates and subcortical volumes in the BPD group (Figure 7B, Supplementary Table S4).

#### 3.4.3. Global Volume Measures

No significant correlations were found between IRDA/IRTA rates and volumes of GM, WM, CSF and cerebellum corrected for the total intracranial volume, respectively, in the BPD group (Figure 7C, Supplementary Table S5).

### 3.5. Correlation with Psychometric Findings

#### 3.5.1. Cortical Thickness

**Figure 8:**
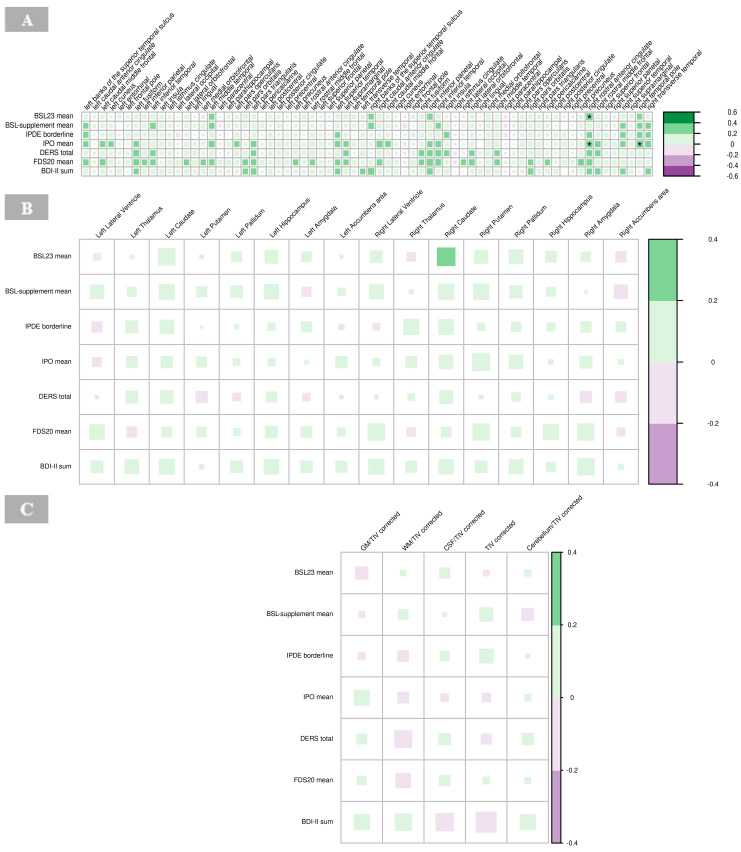
Correlations of psychometric measures with cortical thickness (**A**), subcortical volumes (**B**), and global volumes (gray matter, white matter, cerebrospinal fluid, total intracranial volume, and cerebellum) corrected for total intracranial volume (**C**) in borderline personality disorder. The color of the boxes in the graph represents the direction of the correlation, with green indicating a positive correlation and purple indicating a negative correlation. The size of each box reflects the strength of the correlation, with larger boxes corresponding to stronger correlations with no significant findings (indicated by asterisks as follows: * *p* < 0.05; ** *p* < 0.01; *** *p* < 0.001). Abbreviations: BDI, Beck’s depression inventory; BSL, borderline symptom list; CSF, cerebrospinal fluid; DERS, difficulties in emotion regulation scale; FDS, Freiburger Dissoziationsskala; GM, gray matter; IPDE, International Personality Disorder Examination; IPO, Inventory of Personality Organization; TIV, total intracranial volume; WM, white matter.

There were significant positive associations between BSL23 and IPO with the cortical thickness of the right rostral anterior cingulate cortex (*p =* 0.001), respectively, and IPO scores with the right temporal pole in the BPD group (*p =* 0.001; Figure 8A). High IPO scores, which reflect greater impulsivity and emotional dysregulation, were positively associated with increased cortical thickness in these regions (Figure 8A, Supplementary Table S6).

#### 3.5.2. Subcortical Volumes

No significant correlations were observed between the psychometric scores and subcortical volumes in the BPD group (Figure 8B, Supplementary Table S7).

#### 3.5.3. Global Volumes

No significant associations were found between volumes of GM, WM, CSF, global cerebellum and its ratios with total intracranial volume, respectively, with psychometric findings in the BPD group (Figure 8C, Supplementary Table S8).

### 3.6. Correlation with Neuropsychological Findings

#### 3.6.1. Cortical Thickness

**Figure 9:**
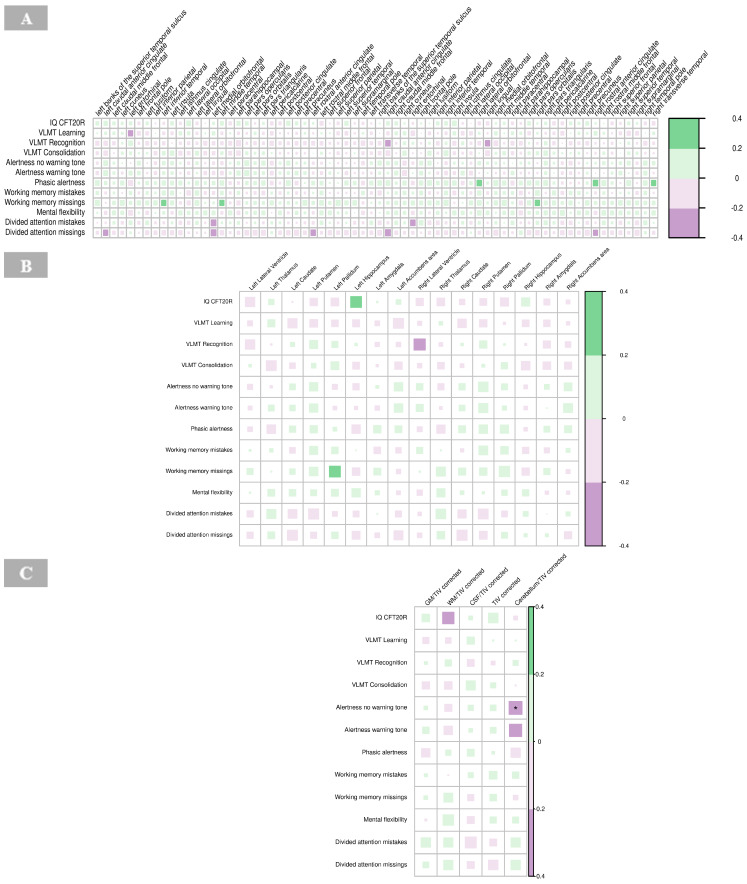
Correlations of neuropsychological tests with cortical thickness (**A**), subcortical volumes (**B**), and global volumes (gray matter, white matter, cerebrospinal fluid, total intracranial volume, and cerebellum) corrected for total intracranial volume (**C**) in borderline personality disorder. The color of the boxes in the graph represents the direction of the correlation, with green indicating a positive correlation and purple indicating a negative correlation. The size of each box reflects the strength of the correlation, with larger boxes corresponding to stronger correlations. Significance is indicated by asterisks as follows: * *p* < 0.05; ** *p* < 0.01; *** *p* < 0.001. Abbreviations: IQ CFT20R, IQ assessed with the Culture Fair Intelligence Test CFT-20-R; CSF, cerebrospinal fluid; GM, gray matter; IQ, intelligence quotient; TIV, total intracranial volume; VLMT, Verbal Learning and Memory Test; WM, white matter.

No significant correlations were revealed for cortical thickness and neuropsychological findings in the BPD group (Figure 9A, Supplementary Table S9).

#### 3.6.2. Subcortical Volumes

No significant correlations were identified between neuropsychological measures and subcortical volumes in the BPD group (Figure 9B, Supplementary Table S10).

#### 3.6.3. Global Volumes

The results revealed a significant negative correlation between alertness (no warning tone; *p* < 0.01) and the global volume of the cerebellum corrected for total intracranial volume in the BPD patient group (Figure 9C, Supplementary Table S11).

## 4. Discussion

A multimodal approach was employed to investigate cerebral structure and associated EEG, as well as psychometric and neuropsychological alterations in BPD. The main findings of this study revealed no significant group differences between BPD and HC in IRDA/IRTA rates before HV. However, the increase in median IRDA/IRTA rates (after versus before HV) was significantly greater in the HC group. Further, no significant group differences were found for quantitative morphometric measures after correcting for multiple testing. Correlation analyses indicated that cortical thinning of the right isthmus of the cingulate gyrus was associated with more pronounced IRDA/IRTA per minute (after versus before HV) in BPD. Also, higher scores on the BSL-23, reflecting increased symptom load, were associated with increased cortical thickness in the right rostral anterior cingulate cortex and higher scores on IPO, representing increased impulsivity, were linked to increased cortical thickness in the right rostral anterior cingulate cortex and right temporal pole. Finally, a significant negative correlation was found between measures of alertness (no warning tone) and cerebellar volume corrected for total intracranial volume.

Group comparisons revealed no IRDA/IRTA differences before HV even though we were actively looking for such patients. There was even a significant larger increase in IRDA/IRTA after HV for the HC group using an automatic quantification approach. This contrasts with previous findings, including our own [35], even though a different method for IRDA/IRTA assessment had been used. This suggests the need for larger studies on the prevalence of EEG pathologies in BPD to resolve these discrepancies. Differences to our earlier study result from stricter inclusion criteria in this study and the automated evaluation approach; in the previous study [35], only a clinical collective was retrospectively analyzed visually, so that organic causalities were less well recorded and no quantitative analyses were performed. Initially, MRI group comparisons revealed several structural abnormalities between BPD patients and HC, including reduced cortical thickness in the right caudal anterior cingulate and significantly smaller total intracranial volume among BPD patients. Further, BPD patients showed larger subcortical volumes in the left and right lateral ventricles, left and right putamen, and right caudate. None of these effects remained significant after correcting for multiple comparisons. This study did not reveal significant alterations in cortical thickness, global as well as subcortical volumes between BPD and HC. These findings contribute to the substantial heterogeneity in the literature reporting morphometric abnormalities in BPD [16,43,44,45,46,47]. Also, the results do not align with the above-mentioned results from meta-analyses. However, methodological differences may be accountable for variation. Importantly, a meta-regression analysis noted that age and medication status of the patient sample moderates functional as well as structural abnormalities [15,17]. Recent attention has been drawn to a transdiagnostic approach, arguing for shared morphometric abnormalities and neural pathways across psychiatric disorders [48,49]. Further, the absence of GM alterations in BPD might be due to the heterogeneity of the disorder with varying symptom profiles, comorbidities, and clinical severities. Such variability may dilute group-level effects, masking structural brain changes that might be evident in more homogenous subgroups (e.g., based on emotional dysregulation or impulsivity). In sum, this highlights the need for nuanced, multimodal approaches to investigate the disorder.

### 4.1. Cingulate Gyrus and IRDA/IRTA Rates

A significant negative correlation between the cortical thickness of the right isthmus of the cingulate gyrus and IRDA/IRTA per minute after versus before HV was detected. Hence, BPD patients with a thinner cortex in the right isthmus cingulate exhibit a greater increase in EEG abnormalities of IRDA/IRTA. The isthmus, located within the posterior cingulate gyrus of the limbic system, connects to the parahippocampal gyrus in the temporal lobe, which is crucial for memory encoding and retrieval [50]. It plays a role in emotional regulation, self-referential processing, and sensory integration, although its full functional scope remains unclear. Previous studies have reported cortical thinning and volumetric changes in both the anterior and posterior cingulate, including the isthmus [44,45,47]. Thinning of the isthmus may suggest impaired neural connectivity or integrity, potentially heightening the susceptibility to stressors, such as HV. Further, it may provoke slow wave activity, IRDA/IRTA, observed in the EEG. Given the isthmus’ involvement in emotional regulation, these structural alterations could underlie paraepileptic dysregulated stress responses and emotional instability seen in individuals with BPD. A pathophysiological explanation could be provided by the local area network inhibition (LANI) hypothesis, according to which slow EEG activity such as IRDA/IRTA in specific brain regions can trigger psychological symptoms by secondary network inhibition [34].

### 4.2. Psychometric Associations with Cortical Thickness

For the BPD patient group, higher scores on the BSL-23, indicating BPD symptom severity, were associated with increased cortical thickness in the right rostral anterior cingulate cortex. Also, the IPO assessing general personality dysfunction with the domain’s identity, defense, and reality testing, were linked to increased cortical thickness in the right rostral anterior cingulate cortex and right temporal pole. The right rostral anterior cingulate cortex is involved in regulating emotional responses, decision-making, and conflict monitoring, while the temporal pole is linked to emotional processing and social cognition [51]. These regions play a crucial role in the regulation of impulsivity and emotional instability—core features of BPD. High IPO scores, which reflect greater impulsivity and emotional dysregulation, were positively associated with increased cortical thickness in these regions. This finding may indicate that structural changes in the right rostral anterior cingulate cortex and temporal pole contribute to the neurobiological basis of impulsive behavior and emotional instability in BPD. The increase in cortical thickness could reflect adaptive or maladaptive neural mechanisms underlying these traits. These findings seem not to be in line with previous research showing reduced volume of the anterior cingulate cortex in BPD adolescents [45,47,52]. Xiao and colleagues [47] found altered surface morphology in the limbic-cortical circuit with reduced mean curvature of the right rostral anterior cingulate cortex in BPD adolescents compared to HC. Overall, dysfunctions of the limbic-cortical circuit have been linked to difficulties in emotion regulation observed in BPD [53], while morphometric alterations of temporal regions have been associated with impaired emotional regulation and deficits in impulse control [47].

### 4.3. Neuropsychological Correlates of Alertness and Attention

With respect to the neuropsychological test performance, there was a significant negative correlation between measures of alertness (no warning tone) and cerebellar volume corrected for total intracranial volume. This finding suggests that larger cerebral volumes relative to total intracranial volume are associated with poorer performance on alertness tasks. It has been well established that the cerebellum predicts and coordinates motor but also cognitive processes as an “internal modeling machine” [54,55,56]. Moreover, the findings resonate with the cerebellar cognitive-affective syndrome framework, derived from lesion analyses, which links cerebellar dysfunction with impairments in executive function, emotional regulation, and attentional processes [57,58,59]. The association between cerebellar volume and reduced alertness performance may imply that cerebellar involvement contributes to attentional and alertness processing in BPD, potentially reflecting compensatory mechanisms or structural adaptations due to attentional demands. Future research should explore the underlying mechanisms driving these relationships, such as potential disruptions in cerebellar-prefrontal networks or maladaptive changes in internal model precision.

### 4.4. Limitations

These findings emphasize the complexity of identifying consistent structural biomarkers in BPD. Given the exploratory nature of this multimodal EEG-MRI study, these results only provide preliminary insights, which should be interpreted with great caution. Further investigation with larger, well-characterized samples is needed to test initial trends and determine genuine neural alterations in BPD. Accounting for high rates of comorbidity in BPD as well as different possible confounding effects such as menstrual cycle phase is crucial and should be considered in future investigations. Ideally, homogeneous patient subgroups (e.g., with dissociations) should be analyzed using such multimodal approaches in the future. It is also important to highlight that this study is methodologically rigorous and, for an exploratory pilot study, based on a relatively large sample size.

## 5. Conclusions

In summary, a link between cerebral changes, EEG alteration, and psychometric as well as neuropsychological findings might be feasible, suggesting that these modalities may capture different neural processes. However, further research is essential to clarify this relationship and to understand its therapeutic implications in clinical populations.

## Figures and Tables

**Table 1:** Comorbid disorders for the borderline personality disorder patient and healthy control group.

Comorbid Disorder	BPD, N = 70 ^1^	HC, N = 36 ^1^
Current or recent depression	62 (85.6%)	0 (0%)
Anxiety disorder	16 (22.9%)	0 (0%)
PTSD	17 (24.9%)	0 (0%)
Autism spectrum disorder	3 (4.4%)	0 (0%)
ADHD	34 (48.6%)	0 (0%)

^1^ n (%; N = N Non-missing). Abbreviations: ADHD, attention-deficit/hyperactivity disorder; BPD, borderline personality disorder; HC, healthy controls; N, sample size; PTSD, post-traumatic stress disorder.

**Table 2:** Psychopharmacological medication in the borderline personality disorder patient and healthy control group.

Status	Psychopharmacological Medication	BPD, N = 70 ^1^	HC, N = 36 ^1^
Current	Antidepressants	46 (65.7%)	0 (0%; N = 36)
	Typical antipsychotics	5 (7.1%)	0 (0%; N = 36)
	Atypical antipsychotics	27 (38.6%)	0 (0%; N = 36)
	Mood stabilizers *	4 (5.7%)	0 (0%; N = 36)
	Benzodiazepine	0 (0%)	0 (0%; N = 36)
	ADHD medication	13 (18.6%)	0 (0%; N = 36)
Former	Antidepressants	48 (68%)	0 (0%; N = 36)
	Typical antipsychotics	8 (11.4%)	0 (0%; N = 36)
	Atypical antipsychotics	28 (40%)	0 (0%; N = 36)
	Mood stabilizer *	6 (8.6%)	0 (0%; N = 36)
	Benzodiazepine	16 (22.9%)	0 (0%; N = 36)
	ADHD medication	12 (17.1%)	0 (0%; N = 36)

* One patient without epilepsy/epileptic seizures was treated with lamotrigine for affective disorder, two patients without epilepsy/epileptic seizures were treated with pregabalin for polyneuropathy and generalized anxiety disorder. ^1^ n (%; N = N Non-missing). Abbreviations: ADHD, attention-deficit/hyperactivity disorder; BPD, borderline personality disorder; HC, healthy controls; N, sample size.

**Table 3:** Psychometry and neuropsychological tests of the borderline personality disorder group and healthy control group.

Psychometry	*p*-Value ^1^	Neuropsychological Test	*p*-Value ^1^
BSL-23	<0.001 ***	CFT-20-R	0.006 **
BSL-Supplement	0.018 **	MWTB	0.452
DERS	<0.001 ***	VLMT Learning	0.017
FDS-20	<0.001 ***	VLMT False positive	0.779
IPO	<0.001 ***	VLMT Preservations	0.444
IPDE Borderline	<0.001 ***	VLMT Recognition	0.421
WURS	<0.001 ***	VLMT Consolidation	0.297
ADHD-Checklist	<0.001 ***	Alertness no warning tone	0.447
BDI-II	<0.001 ***	Alertness warning tone	0.656
EQ	0.003 **	Phasic alertness	0.503
AQ	<0.001 ***	Working memory mistakes	0.249
SCL-90-R Hostility	<0.001 ***	Working memory missing	0.060
SCL-90-R Anxiety	<0.001 ***	Mental flexibility	0.146
SCL-90-R Depression	<0.001 ***	Divided attention mistakes	0.241
SCL-90-R Somatization	<0.001 ***	Divided attention missing	0.020
SCL-90-R Obsessive–compulsive	<0.001 ***		
SCL-90-R Interpersonal sensitivity	<0.001 ***		
SCL-90-R Phobic anxiety	<0.001 ***		
SCL-90-R Paranoid ideation	<0.001 ***		
SCL-90-R Psychoticism	<0.001 ***		
STAI-G Trait	<0.001 ***		

^1^ Wilcoxon rank sum test; Welch Two Sample *t*-test; Fisher’s exact test. ** alpha < 0.01, *** alpha < 0.001 Abbreviations: ADHD, attention-deficit/hyperactivity disorder; AQ, Autism Spectrum Quotient; BDI-II, Beck Depression Inventory II; BSL, Borderline-Symptom List; CFT-20-R, Culture Fair Intelligence Test; DERS, Difficulties in Emotion Regulation Scale; EQ, Cambridge Behaviour Scale-40; FDS-20, Dissociation Experience Scale; MWTB, Multiple-choice Word Test; *p*-value, probability value; SCL-90-R, Symptom-Checklist; STAI-G, State-Trait Anxiety Inventory; VLMT, Verbal Learning and Memory Test; WURS, Wender Utah Rating Scale.

## Data Availability

The original contributions presented in this study are included in the article and Supplementary Materials. Further inquiries can be directed to the corresponding author.

## Supplementary Materials

The following supporting information can be downloaded at: https://www.mdpi.com/article/10.3390/brainsci15060592/s1, Table S1: Psychometry and neuropsychological test battery for the borderline personality disorder patient and healthy control group; Table S2: Sociodemographic characteristics of the borderline personality disorder and healthy control group; Table S3: Correlations of electroencephalography findings and cortical thickness of patients with borderline personality disorder; Table S4: Correlations of electroencephalography findings and subcortical volumes of patients with borderline personality disorder; Table S5: Correlations of electroencephalography findings and global volume measures of patients with borderline personality disorder; Table S6: Correlations of psychometric findings and cortical thickness of patients with borderline personality disorder; Table S7: Correlations of psychometric findings and subcortical volumes of patients with borderline personality disorder; Table S8: Correlations of psychometric findings and global volume measures of patients with borderline personality disorder; Table S9: Correlations of neuropsychological findings and cortical thickness of patients with borderline personality disorder; Table S10: Correlations of neuropsychological findings and subcortical volumes of patients with borderline personality disorder; Table S11: Correlations of neuropsychological findings and global volume measures of patients with borderline personality disorder.
