# Supplementary material for: Brain Structural Correlates of EEG Network Hyperexcitability, Symptom Severity, Attention, and Memory in Borderline Personality Disorder
Source: Brain Sci. 2025 May 31;15(6):592. doi: 10.3390/brainsci15060592 (PMC12191120; doi:10.3390/brainsci15060592)
Supplement: Supplementary file 1 [file brainsci-15-00592-s001.zip › brainsci-3608228-supplementary.pdf]

Supplementary materials

Questionnaire	Reference
Structured Clinical Interview for DSM-IV (SCID-I/SCID-II)	First et al., 1997 [1]
Borderline-Symptom List (BSL)	Bohus et al., 2001 [2]
Dissociation Experience Scale (FDS-20)	Freyberger et al., 1998 [3]
Symptom-Checklist (SCL-90-R)	Derogatis et al., 1976 [4]
Beck Depression Inventory II (BDI-II)	Hautzinger et al., 2006 [5]
State-Trait-Anxiety-Inventory (STAI-G)	Laux et al., 1981 [6]
Wender Utah Rating Scale (WURS)	Retz-Junginger et al. 2002 [7]
ADHD-Checklist for DSM-IV (ADHD-CL)	Rösler et al. 2004 [8]
Autism Spectrum Quotient (AQ)	Baron-Cohen et al., 2001 [9]
Cambridge Behaviour Scale-40 (Empathy Quotient)	Baron-Cohen & Wheelwright, 2004 [10]
Inventory Of Personality Organization (IPO-16)	Clarkin et al., 2001 [11]
Difficulties in Emotion Regulation Scale (DERS)	Gratz & Roemer, 2004 [12]
**Neuropsychological Test**	**Reference**
Test for Attentional Performances (TAP)	Zimmermann & Fimm, 1993 [13]
Verbal Learning and Memory Test (VLMT)	Helmstaedter et al., 1990 [14]
Culture Fair Intelligence Test (CFT-20 R)	Weiß, 2019 [15]
Multiple-choice word test (MWT-B)	Lehrl et al., 1995 [16]

Supplementary Table S1: Psychometry and neuropsychological test battery for the borderline personality disorder patient and healthy control group. Abbreviations: ADHD-CL, ADHD-Checklist for DSM-IV; AQ, Autism Spectrum Quotient; BDI-II, Beck Depression Inventory II; BSL, Borderline-Symptom List; CFT-20 R, Culture Fair Intelligence Test; DERS, Difficulties in Emotion Regulation Scale; EQ, Cambridge Behaviour Scale-40; FDS-20, Dissociation Experience Scale; IPO-16, Inventory of Personality Organization; MWT-B, Multiple-choice Word Test; SCL-90-R, Symptom-Checklist; SCID-I/SCID-II, Structured Clinical Interview for DSM-IV; STAI-G, State-Trait Anxiety Inventory; TAP, Test for Attentional Performances; VLMT, Verbal Learning and Memory Test; WURS, Wender Utah Rating Scale.

References

1. First, M.B.; Spitzer, R.L.; Gibbon, M.; Williams, J.B.W. *Structured Clinical Interview for DSM-IV Axis I Disorders (SCID-I) and Axis II Disorders (SCID-II)*; American Psychiatric Press: Washington, DC, USA, 1997;
2. Bohus, M.; Limberger, M.F.; Sender, I.; Gratwohl, T.; Stieglitz, R.-D. Entwicklung Der Borderline-Symptom-Liste (Development of Borderline Symptom List). *Psychother. Psychosom. Med. Psychol.* **2001**, *51*, 201–211, doi:10.1055/s-2001-13281.
3. Freyberger, H.J.; Spitzer, C.; Stieglitz, R.-D.; Kuhn, G.; Magdeburg, N.; Bernstein-Carlson, E. Fragebogen Zu Dissoziativen Symptomen (FDS). Deutsche Adaptation, Reliabilität Und Validität Der Amerikanischen Dissociative Experience Scale (DES). [The Fragebogen (Questionnaire) Zu Dissoziativen Symptomen (FDS): German Adaptation, Reliability, and Validity of the American Dissociative Experience Scale (DES).]. *PPmP Psychother. Psychosom. Med. Psychol.* **1998**, *48*, 223–229.
4. Derogatis, L.R. *The Symptom Checklist-90-R (SCL-90-R): Administration, Scoring, and Procedures Manual*; Clinical Psychometric Research: Baltimore, MD, USA, 1976;
5. Hautzinger, M.; Keller, F.; Kühner, C. *Beck Depressions-Inventar Revision (BDI-II)*; Harcourt Test Services: Frankfurt am Main, Germany, 2006;
6. Laux, L.; Glanzmann, P.; Schaffner, P.; Spielberger, C.D. *Das State-Trait-Angstinventar (STAI): Theoretische Grundlagen Und Handanweisung*; Beltz: Weinheim, Germany, 1981;
7. Retz-Junginger, P.; Retz, W.; Blocher, D.; Weijers, H.-G.; Trott, G.-E.; Wender, P.H.; Rössler, M. Wender Utah Rating Scale (WURS-k) Die deutsche Kurzform zur retrospektiven Erfassung des hyperkinetischen Syndroms bei Erwachsenen. *Nervenarzt* **2002**, *73*, 830–838, doi:10.1007/s00115-001-1215-x.
8. Rösler, M.; Retz, W.; Retz-Junginger, P.; Thome, J.; Supprian, T.; Nissen, T.; Stieglitz, R.-D.; Blocher, D.; Hengesch, G.; Trott, G.E. Instrumente zur Diagnostik der Aufmerksamkeitsdefizit-/Hyperaktivitätsstörung (ADHS) im Erwachsenenalter. *Nervenarzt* **2004**, *75*, 888–895, doi:10.1007/s00115-003-1622-2.
9. Baron-Cohen, S.; Wheelwright, S.; Skinner, R.; Martin, J.; Clubley, E. The Autism-Spectrum Quotient (AQ): Evidence from Asperger Syndrome/High-Functioning Autism, Males and Females, Scientists and Mathematicians. *J. Autism Dev. Disord.* **2001**, *31*, 5–17, doi:10.1023/a:1005653411471.
10. Baron-Cohen, S.; Wheelwright, S. The Empathy Quotient: An Investigation of Adults with Asperger Syndrome or High Functioning Autism, and Normal Sex Differences. *J. Autism Dev. Disord.* **2004**, *34*, 163–175, doi:10.1023/b:jadd.0000022607.19833.00.
11. Clarkin, J.F.; Foelsch, P.A.; Kernberg, O.F.; Ingenhoven, T. THE INVENTORY OF PERSONALITY ORGANISATION. **2001**.
12. Gratz, K.L.; Roemer, L. Multidimensional Assessment of Emotion Regulation and Dysregulation: Development, Factor Structure, and Initial Validation of the Difficulties in Emotion Regulation Scale. *J. Psychopathol. Behav. Assess.* **2004**, *26*, 41–54, doi:10.1023/B:JOBA.0000007455.08539.94.
13. Zimmermann, P.; Fimm, B. *Testbatterie Zur Aufmerksamkeitsprüfung (TAP)*; Psytest: Herzogenrath, Germany, 1993;
14. Helmstaedter, C.; Durwen, H.F. VLMT: Verbaler Lern- Und Merkfähigkeitstest: Ein Praktikables Und Differenziertes Instrumentarium Zur Prüfung Der Verbalen Gedächtnisleistungen. [VLMT: A Useful Tool to Assess and Differentiate Verbal Memory Performance.]. *Schweiz. Arch. Für Neurol. Neurochir. Psychiatr.* **1990**, *141*, 21–30.
15. Weiß, R. CFT 20-R Testheft, 2. Auflage Kaufen | Hogrefe-Verlag | SpielundLern Available online: https://www.spielundlern.de/product_info.php/products_id/195230 (accessed on 29 May 2024).

16. Lehrl, S.; Triebig, G.; Fischer, B. Multiple Choice Vocabulary Test MWT as a Valid and Short Test to Estimate Premorbid Intelligence. *Acta Neurol. Scand.* **1995**, *91*, 335–345, doi:10.1111/j.1600-0404.1995.tb07018.x.

Sociodemographic characteristic	BPD, N = 70[1]	HC, N = 36[1]	p-value[2]
Age	30.14 ± 9.5 (N = 70)	27.08 ± 7.4 (N = 36)	0.071
Education level			<0.001
University degree	9 (13%; N = 67)	18 (51%; N = 35)	
High degree	25 (37%; N = 67)	15 (43%; N = 35)	
Middle degree	27 (40%; N = 67)	2 (5.7%; N = 35)	
Low degree	5 (7.5%; N = 67)	0 (0%; N = 35)	
Other qualification	1 (1.5%; N = 67)	0 (0%; N = 35)	
Employment status			<0.001
Unemployed	24 (36%; N = 67)	0 (0%; N = 35)	
Retired	6 (9.0%; N = 67)	0 (0%; N = 35)	
Apprentice	2 (3.0%; N = 67)	2 (5.7%; N = 35)	
Student	10 (15%; N = 67)	20 (57%; N = 35)	
Part-time job	21 (31%; N = 67)	7 (20%; N = 35)	
Full-time job	4 (6.0%; N = 67)	6 (17%; N = 35)	
Marital status			0.435
Single/ unwed	57 (85%; N = 67)	33 (94%; N = 35)	
Married	5 (7.5%; N = 67)	1 (2.9%; N = 35)	
Divorced	5 (7.5%; N = 67)	1 (2.9%; N = 35)	
Handedness			0.106
Both	4 (6.0%; N = 67)	0 (0%; N = 35)	
Left	5 (7.5%; N = 67)	0 (0%; N = 35)	
Right	58 (87%; N = 67)	35 (100%; N = 35)	
BMI	27.0 ± 7.3 (N = 70)	21.4 ± 1.9 (N = 36)	<0.001
Tobacco	27 (40%; N = 68)	7 (19%; N = 36)	0.048
Package years	2.88 ± 6.3 (N = 65)	0.31 ± 0.9 (N = 36)	0.002

Supplementary Table S2. Sociodemographic characteristics of the borderline personality disorder and healthy control group. [1]Mean ± SD; n (%; N = N Non-missing. [2]Welch Two Sample t-test; Fisher's exact test. Abbreviations: BMI, body mass-index; BPD, borderline personality disorder; HC, healthy controls; N, sample size; p-value, probability value; SD, standard deviation.

EEG (IRDA/IRTA)	Brain Region	r	p-value	adjusted p-value
IRDA/IRTA per min before HV	left banks of the superior temporal sulcus	0.089	0.464	0.585
IRDA/IRTA per min before HV	left caudal anterior cingulate	-0.037	0.761	0.822
IRDA/IRTA per min before HV	left caudal middle frontal	0.229	0.057	0.185
IRDA/IRTA per min before HV	left cuneus	0.186	0.123	0.245
IRDA/IRTA per min before HV	left entorhinal	0.017	0.890	0.931
IRDA/IRTA per min before HV	left frontal pole	0.139	0.251	0.388
IRDA/IRTA per min before HV	left fusiform	0.227	0.059	0.185
IRDA/IRTA per min before HV	left inferior parietal	0.303	0.011	0.185
IRDA/IRTA per min before HV	left inferior temporal	0.156	0.197	0.319
IRDA/IRTA per min before HV	left insula	0.143	0.238	0.377
IRDA/IRTA per min before HV	left isthmus cingulate	0.052	0.668	0.769
IRDA/IRTA per min before HV	left lateral occipital	0.044	0.716	0.798
IRDA/IRTA per min before HV	left lateral orbitofrontal	0.241	0.045	0.185
IRDA/IRTA per min before HV	left lingual	0.226	0.060	0.185
IRDA/IRTA per min before HV	left medial orbitofrontal	0.096	0.429	0.550
IRDA/IRTA per min before HV	left middle temporal	0.121	0.318	0.451
IRDA/IRTA per min before HV	left paracentral	0.219	0.069	0.193
IRDA/IRTA per min before HV	left parahippocampal	-0.008	0.946	0.955
IRDA/IRTA per min before HV	left pars opercularis	0.050	0.679	0.769
IRDA/IRTA per min before HV	left pars orbitalis	0.107	0.379	0.514
IRDA/IRTA per min before HV	left pars triangularis	0.183	0.130	0.245
IRDA/IRTA per min before HV	left pericalcarine	0.323	0.006	0.185
IRDA/IRTA per min before HV	left postcentral	0.293	0.014	0.185
IRDA/IRTA per min before HV	left posterior cingulate	0.184	0.128	0.245
IRDA/IRTA per min before HV	left precentral	0.263	0.028	0.185
IRDA/IRTA per min before HV	left precuneus	0.279	0.019	0.185
IRDA/IRTA per min before HV	left rostral anterior cingulate	0.179	0.139	0.245
IRDA/IRTA per min before HV	left rostral middle frontal	0.194	0.108	0.244
IRDA/IRTA per min before HV	left superior frontal	0.179	0.139	0.245
IRDA/IRTA per min before HV	left superior parietal	0.243	0.043	0.185
IRDA/IRTA per min before HV	left superior temporal	0.176	0.145	0.247
IRDA/IRTA per min before HV	left supramarginal	0.190	0.114	0.245
IRDA/IRTA per min before HV	left temporal pole	0.008	0.945	0.955

IRDA/IRTA per min before HV	left transverse temporal	0.236	0.050	0.185
IRDA/IRTA per min before HV	right banks of the superior temporal sulcus	0.072	0.556	0.687
IRDA/IRTA per min before HV	right caudal anterior cingulate	0.105	0.389	0.514
IRDA/IRTA per min before HV	right caudal middle frontal	0.137	0.257	0.388
IRDA/IRTA per min before HV	right cuneus	0.158	0.191	0.317
IRDA/IRTA per min before HV	right entorhinal	0.007	0.955	0.955
IRDA/IRTA per min before HV	right frontal pole	0.104	0.393	0.514
IRDA/IRTA per min before HV	right fusiform	0.323	0.006	0.185
IRDA/IRTA per min before HV	right inferior parietal	0.228	0.058	0.185
IRDA/IRTA per min before HV	right inferior temporal	0.274	0.022	0.185
IRDA/IRTA per min before HV	right insula	0.056	0.643	0.754
IRDA/IRTA per min before HV	right isthmus cingulate	0.214	0.076	0.196
IRDA/IRTA per min before HV	right lateral occipital	0.256	0.032	0.185
IRDA/IRTA per min before HV	right lateral orbitofrontal	0.272	0.023	0.185
IRDA/IRTA per min before HV	right lingual	0.217	0.071	0.193
IRDA/IRTA per min before HV	right medial orbitofrontal	-0.019	0.876	0.931
IRDA/IRTA per min before HV	right middle temporal	0.239	0.046	0.185
IRDA/IRTA per min before HV	right paracentral	0.189	0.116	0.245
IRDA/IRTA per min before HV	right parahippocampal	-0.061	0.614	0.745
IRDA/IRTA per min before HV	right pars opercularis	0.267	0.025	0.185
IRDA/IRTA per min before HV	right pars orbitalis	0.203	0.092	0.224
IRDA/IRTA per min before HV	right pars triangularis	0.187	0.121	0.245
IRDA/IRTA per min before HV	right pericalcarine	0.224	0.063	0.185
IRDA/IRTA per min before HV	right postcentral	0.225	0.061	0.185
IRDA/IRTA per min before HV	right posterior cingulate	0.115	0.344	0.478
IRDA/IRTA per min before HV	right precentral	0.247	0.039	0.185
IRDA/IRTA per min before HV	right precuneus	0.257	0.032	0.185
IRDA/IRTA per min before HV	right rostral anterior cingulate	0.060	0.624	0.745
IRDA/IRTA per min before HV	right rostral middle frontal	0.178	0.140	0.245
IRDA/IRTA per min before HV	right superior frontal	0.123	0.311	0.451
IRDA/IRTA per min before HV	right superior parietal	0.212	0.078	0.196
IRDA/IRTA per min before HV	right superior temporal	0.195	0.105	0.244
IRDA/IRTA per min before HV	right supramarginal	0.228	0.058	0.185
IRDA/IRTA per min before HV	right temporal pole	0.037	0.759	0.822
IRDA/IRTA per min before HV	right transverse temporal	0.130	0.283	0.418

IRDA/IRTA per min before vs. after HV	left banks of the superior temporal sulcus	-0.033	0.792	0.880
IRDA/IRTA per min before vs. after HV	left caudal anterior cingulate	0.071	0.568	0.721
IRDA/IRTA per min before vs. after HV	left caudal middle frontal	-0.251	0.041	0.296
IRDA/IRTA per min before vs. after HV	left cuneus	-0.122	0.325	0.491
IRDA/IRTA per min before vs. after HV	left entorhinal	0.191	0.122	0.320
IRDA/IRTA per min before vs. after HV	left frontal pole	-0.044	0.721	0.859
IRDA/IRTA per min before vs. after HV	left fusiform	-0.124	0.316	0.491
IRDA/IRTA per min before vs. after HV	left inferior parietal	-0.228	0.063	0.307
IRDA/IRTA per min before vs. after HV	left inferior temporal	-0.200	0.105	0.317
IRDA/IRTA per min before vs. after HV	left insula	-0.011	0.928	0.962
IRDA/IRTA per min before vs. after HV	left isthmus cingulate	-0.268	0.028	0.274
IRDA/IRTA per min before vs. after HV	left lateral occipital	-0.118	0.340	0.502
IRDA/IRTA per min before vs. after HV	left lateral orbitofrontal	-0.136	0.271	0.460
IRDA/IRTA per min before vs. after HV	left lingual	-0.128	0.301	0.487
IRDA/IRTA per min before vs. after HV	left medial orbitofrontal	-0.201	0.102	0.317
IRDA/IRTA per min before vs. after HV	left middle temporal	-0.164	0.184	0.356
IRDA/IRTA per min before vs. after HV	left paracentral	-0.106	0.395	0.572
IRDA/IRTA per min before vs. after HV	left parahippocampal	0.167	0.176	0.353
IRDA/IRTA per min before vs. after HV	left pars opercularis	-0.229	0.063	0.307
IRDA/IRTA per min before vs. after HV	left pars orbitalis	-0.185	0.134	0.328
IRDA/IRTA per min before vs. after HV	left pars triangularis	-0.259	0.034	0.292
IRDA/IRTA per min before vs. after HV	left pericalcarine	-0.027	0.827	0.892
IRDA/IRTA per min before vs. after HV	left postcentral	-0.197	0.110	0.317
IRDA/IRTA per min before vs. after HV	left posterior cingulate	-0.061	0.624	0.772
IRDA/IRTA per min before vs. after HV	left precentral	-0.195	0.113	0.317
IRDA/IRTA per min before vs. after HV	left precuneus	-0.167	0.176	0.353
IRDA/IRTA per min before vs. after HV	left rostral anterior cingulate	-0.247	0.044	0.296
IRDA/IRTA per min before vs. after HV	left rostral middle frontal	-0.303	0.013	0.274
IRDA/IRTA per min before vs. after HV	left superior frontal	-0.218	0.076	0.317
IRDA/IRTA per min before vs. after HV	left superior parietal	-0.168	0.173	0.353
IRDA/IRTA per min before vs. after HV	left superior temporal	-0.001	0.996	0.996
IRDA/IRTA per min before vs. after HV	left supramarginal	-0.176	0.155	0.353
IRDA/IRTA per min before vs. after HV	left temporal pole	-0.031	0.802	0.880
IRDA/IRTA per min before vs. after HV	left transverse temporal	0.005	0.965	0.980
IRDA/IRTA per min before vs. after HV	right banks of the superior temporal sulcus	-0.194	0.116	0.317

IRDA/IRTA per min before vs. after HV	right caudal anterior cingulate	-0.077	0.537	0.703
IRDA/IRTA per min before vs. after HV	right caudal middle frontal	-0.200	0.105	0.317
IRDA/IRTA per min before vs. after HV	right cuneus	-0.042	0.733	0.859
IRDA/IRTA per min before vs. after HV	right entorhinal	0.049	0.692	0.840
IRDA/IRTA per min before vs. after HV	right frontal pole	-0.070	0.573	0.721
IRDA/IRTA per min before vs. after HV	right fusiform	-0.163	0.189	0.356
IRDA/IRTA per min before vs. after HV	right inferior parietal	-0.212	0.085	0.317
IRDA/IRTA per min before vs. after HV	right inferior temporal	-0.234	0.057	0.307
IRDA/IRTA per min before vs. after HV	right insula	-0.122	0.324	0.491
IRDA/IRTA per min before vs. after HV	right isthmus cingulate	-0.419	<0.001	0.028
IRDA/IRTA per min before vs. after HV	right lateral occipital	-0.202	0.101	0.317
IRDA/IRTA per min before vs. after HV	right lateral orbitofrontal	-0.275	0.024	0.274
IRDA/IRTA per min before vs. after HV	right lingual	-0.236	0.054	0.307
IRDA/IRTA per min before vs. after HV	right medial orbitofrontal	-0.206	0.095	0.317
IRDA/IRTA per min before vs. after HV	right middle temporal	-0.286	0.019	0.274
IRDA/IRTA per min before vs. after HV	right paracentral	-0.077	0.537	0.703
IRDA/IRTA per min before vs. after HV	right parahippocampal	0.038	0.759	0.864
IRDA/IRTA per min before vs. after HV	right pars opercularis	-0.225	0.068	0.307
IRDA/IRTA per min before vs. after HV	right pars orbitalis	-0.184	0.135	0.328
IRDA/IRTA per min before vs. after HV	right pars triangularis	-0.286	0.019	0.274
IRDA/IRTA per min before vs. after HV	right pericalcarine	-0.038	0.763	0.864
IRDA/IRTA per min before vs. after HV	right postcentral	-0.149	0.228	0.397
IRDA/IRTA per min before vs. after HV	right posterior cingulate	-0.014	0.913	0.962
IRDA/IRTA per min before vs. after HV	right precentral	-0.132	0.288	0.478
IRDA/IRTA per min before vs. after HV	right precuneus	-0.151	0.221	0.396
IRDA/IRTA per min before vs. after HV	right rostral anterior cingulate	-0.100	0.422	0.598
IRDA/IRTA per min before vs. after HV	right rostral middle frontal	-0.275	0.024	0.274
IRDA/IRTA per min before vs. after HV	right superior frontal	-0.169	0.171	0.353
IRDA/IRTA per min before vs. after HV	right superior parietal	-0.097	0.437	0.606
IRDA/IRTA per min before vs. after HV	right superior temporal	-0.010	0.934	0.962
IRDA/IRTA per min before vs. after HV	right supramarginal	-0.155	0.211	0.388
IRDA/IRTA per min before vs. after HV	right temporal pole	-0.077	0.537	0.703
IRDA/IRTA per min before vs. after HV	right transverse temporal	-0.173	0.162	0.353

Supplementary Table S3. Correlations of electroencephalography findings and cortical thickness of patients with borderline personality disorder. Abbreviations: *HV, hyperventilation; IRDA/IRTA, intermittent rhythmic delta/theta activity.*

EEG (IRDA/IRTA)	Brain Region	r	p-value	adjusted p-value
IRDA/IRTA per min before HV	Left Lateral Ventricle	0.050	0.681	0.759
IRDA/IRTA per min before HV	Left Thalamus	0.072	0.555	0.718
IRDA/IRTA per min before HV	Left Caudate	0.196	0.104	0.330
IRDA/IRTA per min before HV	Left Putamen	0.186	0.124	0.330
IRDA/IRTA per min before HV	Left Pallidum	0.130	0.285	0.507
IRDA/IRTA per min before HV	Left Hippocampus	0.155	0.201	0.402
IRDA/IRTA per min before HV	Left Amygdala	0.067	0.583	0.718
IRDA/IRTA per min before HV	Left Accumbens area	-0.045	0.711	0.759
IRDA/IRTA per min before HV	Right Lateral Ventricle	0.068	0.577	0.718
IRDA/IRTA per min before HV	Right Thalamus	0.214	0.076	0.330
IRDA/IRTA per min before HV	Right Caudate	0.202	0.093	0.330
IRDA/IRTA per min before HV	Right Putamen	0.164	0.174	0.398
IRDA/IRTA per min before HV	Right Pallidum	0.205	0.089	0.330
IRDA/IRTA per min before HV	Right Hippocampus	-0.003	0.978	0.978
IRDA/IRTA per min before HV	Right Amygdala	0.205	0.089	0.330
IRDA/IRTA per min before HV	Right Accumbens area	-0.109	0.368	0.589
IRDA/IRTA per min before vs. after HV	Left Lateral Ventricle	-0.028	0.820	0.959
IRDA/IRTA per min before vs. after HV	Left Thalamus	-0.006	0.959	0.959
IRDA/IRTA per min before vs. after HV	Left Caudate	0.055	0.659	0.959
IRDA/IRTA per min before vs. after HV	Left Putamen	-0.014	0.908	0.959
IRDA/IRTA per min before vs. after HV	Left Pallidum	-0.175	0.157	0.677
IRDA/IRTA per min before vs. after HV	Left Hippocampus	0.258	0.035	0.313
IRDA/IRTA per min before vs. after HV	Left Amygdala	0.034	0.786	0.959
IRDA/IRTA per min before vs. after HV	Left Accumbens area	0.170	0.169	0.677
IRDA/IRTA per min before vs. after HV	Right Lateral Ventricle	0.045	0.717	0.959
IRDA/IRTA per min before vs. after HV	Right Thalamus	-0.058	0.639	0.959
IRDA/IRTA per min before vs. after HV	Right Caudate	0.037	0.765	0.959
IRDA/IRTA per min before vs. after HV	Right Putamen	-0.008	0.946	0.959
IRDA/IRTA per min before vs. after HV	Right Pallidum	-0.109	0.379	0.959
IRDA/IRTA per min before vs. after HV	Right Hippocampus	0.253	0.039	0.313
IRDA/IRTA per min before vs. after HV	Right Amygdala	0.092	0.457	0.959
IRDA/IRTA per min before vs. after HV	Right Accumbens area	0.043	0.731	0.959

Supplementary Table S4. Correlations of electroencephalography findings and subcortical volumes of patients with borderline personality disorder. Abbreviations: *HV, hyperventilation; IRDA/IRTA, intermittent rhythmic delta/theta activity.*

EEG (IRDA/IRTA)	Brain Region	r	p-value	adjusted p-value
IRDA/IRTA per min before HV	GM/TIV corrected	0.105	0.389	0.705
IRDA/IRTA per min before HV	WM/TIV corrected	0.028	0.820	0.820
IRDA/IRTA per min before HV	CSF/TIV corrected	-0.070	0.564	0.705
IRDA/IRTA per min before HV	TIV corrected	0.141	0.244	0.705
IRDA/IRTA per min before HV	Cerebellum/TIV corrected	0.070	0.563	0.705
IRDA/IRTA per min before vs. after HV	GM/TIV corrected	-0.078	0.528	0.837
IRDA/IRTA per min before vs. after HV	WM/TIV corrected	-0.026	0.837	0.837
IRDA/IRTA per min before vs. after HV	CSF/TIV corrected	0.085	0.495	0.837
IRDA/IRTA per min before vs. after HV	TIV corrected	0.114	0.359	0.837
IRDA/IRTA per min before vs. after HV	Cerebellum/TIV corrected	-0.028	0.820	0.837

Supplementary Table S5. Correlations of electroencephalography findings and global volume measures of patients with borderline personality disorder. Abbreviations: *CSF, cerebrospinal fluid; GM, grey matter; HV, hyperventilation; IRDA/IRTA, intermittent rhythmic delta/theta activity; TIV, total intracranial volume; WM, white matter.*

Questionnaire	Brain Region	r	p-value	Adjusted p-value
BSL23 mean	left banks of the superior temporal sulcus	0.163	0.184	0.482
BSL23 mean	left caudal anterior cingulate	0.074	0.547	0.702
BSL23 mean	left caudal middle frontal	0.154	0.209	0.486
BSL23 mean	left cuneus	0.059	0.634	0.757
BSL23 mean	left entorhinal	0.131	0.286	0.518
BSL23 mean	left frontal pole	0.082	0.509	0.665
BSL23 mean	left fusiform	0.198	0.106	0.424
BSL23 mean	left inferior parietal	0.158	0.198	0.486
BSL23 mean	left inferior temporal	0.201	0.100	0.424
BSL23 mean	left insula	0.164	0.181	0.482
BSL23 mean	left isthmus cingulate	0.117	0.342	0.542
BSL23 mean	left lateral occipital	-0.013	0.918	0.931
BSL23 mean	left lateral orbitofrontal	0.146	0.235	0.515
BSL23 mean	left lingual	-0.033	0.789	0.852
BSL23 mean	left medial orbitofrontal	0.126	0.304	0.518
BSL23 mean	left middle temporal	0.280	0.021	0.424
BSL23 mean	left paracentral	-0.015	0.902	0.931
BSL23 mean	left parahippocampal	0.101	0.411	0.585
BSL23 mean	left pars opercularis	0.126	0.305	0.518
BSL23 mean	left pars orbitalis	0.218	0.074	0.424
BSL23 mean	left pars triangularis	0.195	0.112	0.424
BSL23 mean	left pericalcarine	0.009	0.945	0.945
BSL23 mean	left postcentral	0.154	0.210	0.486
BSL23 mean	left posterior cingulate	0.119	0.335	0.542
BSL23 mean	left precentral	0.065	0.599	0.741

BSL23 mean	left precuneus	0.105	0.396	0.585
BSL23 mean	left rostral anterior cingulate	0.189	0.122	0.424
BSL23 mean	left rostral middle frontal	0.120	0.328	0.542
BSL23 mean	left superior frontal	0.061	0.621	0.754
BSL23 mean	left superior parietal	0.054	0.664	0.779
BSL23 mean	left superior temporal	0.181	0.139	0.424
BSL23 mean	left supramarginal	0.194	0.113	0.424
BSL23 mean	left temporal pole	0.179	0.143	0.424
BSL23 mean	left transverse temporal	0.141	0.251	0.518
BSL23 mean	right banks of the superior temporal sulcus	0.262	0.031	0.424
BSL23 mean	right caudal anterior cingulate	0.231	0.058	0.424
BSL23 mean	right caudal middle frontal	0.109	0.375	0.580
BSL23 mean	right cuneus	0.044	0.723	0.806
BSL23 mean	right entorhinal	-0.037	0.762	0.836
BSL23 mean	right frontal pole	0.132	0.284	0.518
BSL23 mean	right fusiform	0.202	0.099	0.424
BSL23 mean	right inferior parietal	0.290	0.016	0.424
BSL23 mean	right inferior temporal	0.244	0.045	0.424
BSL23 mean	right insula	0.082	0.509	0.665
BSL23 mean	right isthmus cingulate	-0.049	0.690	0.795
BSL23 mean	right lateral occipital	-0.044	0.721	0.806
BSL23 mean	right lateral orbitofrontal	0.152	0.215	0.486
BSL23 mean	right lingual	-0.014	0.912	0.931
BSL23 mean	right medial orbitofrontal	0.249	0.040	0.424
BSL23 mean	right middle temporal	0.185	0.132	0.424
BSL23 mean	right paracentral	-0.097	0.429	0.595
BSL23 mean	right parahippocampal	0.028	0.822	0.873
BSL23 mean	right pars opercularis	0.138	0.263	0.518

BSL23 mean	right pars orbitalis	0.181	0.139	0.424
BSL23 mean	right pars triangularis	0.130	0.289	0.518
BSL23 mean	right pericalcarine	0.131	0.286	0.518
BSL23 mean	right postcentral	0.139	0.260	0.518
BSL23 mean	right posterior cingulate	0.068	0.583	0.735
BSL23 mean	right precentral	0.082	0.508	0.665
BSL23 mean	right precuneus	0.163	0.184	0.482
BSL23 mean	right rostral anterior cingulate	0.411	<0.001	0.034
BSL23 mean	right rostral middle frontal	0.222	0.069	0.424
BSL23 mean	right superior frontal	0.107	0.384	0.581
BSL23 mean	right superior parietal	0.101	0.413	0.585
BSL23 mean	right superior temporal	0.201	0.101	0.424
BSL23 mean	right supramarginal	0.185	0.130	0.424
BSL23 mean	right temporal pole	0.257	0.034	0.424
BSL23 mean	right transverse temporal	0.186	0.130	0.424
BSL-supplement mean	left banks of the superior temporal sulcus	0.258	0.034	0.314
BSL-supplement mean	left caudal anterior cingulate	0.114	0.355	0.527
BSL-supplement mean	left caudal middle frontal	0.164	0.182	0.390
BSL-supplement mean	left cuneus	0.125	0.310	0.517
BSL-supplement mean	left entorhinal	-0.004	0.974	0.988
BSL-supplement mean	left frontal pole	0.061	0.623	0.757
BSL-supplement mean	left fusiform	0.149	0.225	0.437
BSL-supplement mean	left inferior parietal	0.174	0.156	0.390
BSL-supplement mean	left inferior temporal	0.309	0.010	0.314
BSL-supplement mean	left insula	0.204	0.095	0.378
BSL-supplement mean	left isthmus cingulate	0.161	0.190	0.390
BSL-supplement mean	left lateral occipital	0.026	0.834	0.915
BSL-supplement mean	left lateral orbitofrontal	0.161	0.189	0.390

BSL-supplement mean	left lingual	0.128	0.300	0.517
BSL-supplement mean	left medial orbitofrontal	0.201	0.100	0.378
BSL-supplement mean	left middle temporal	0.294	0.015	0.314
BSL-supplement mean	left paracentral	-0.061	0.623	0.757
BSL-supplement mean	left parahippocampal	0.176	0.150	0.390
BSL-supplement mean	left pars opercularis	0.127	0.302	0.517
BSL-supplement mean	left pars orbitalis	0.155	0.208	0.416
BSL-supplement mean	left pars triangularis	0.248	0.042	0.314
BSL-supplement mean	left pericalcarine	0.169	0.168	0.390
BSL-supplement mean	left postcentral	0.045	0.715	0.824
BSL-supplement mean	left posterior cingulate	0.161	0.188	0.390
BSL-supplement mean	left precentral	0.020	0.870	0.939
BSL-supplement mean	left precuneus	0.089	0.470	0.641
BSL-supplement mean	left rostral anterior cingulate	0.046	0.710	0.824
BSL-supplement mean	left rostral middle frontal	0.114	0.357	0.527
BSL-supplement mean	left superior frontal	0.068	0.580	0.735
BSL-supplement mean	left superior parietal	-0.009	0.944	0.972
BSL-supplement mean	left superior temporal	0.175	0.154	0.390
BSL-supplement mean	left supramarginal	0.121	0.327	0.517
BSL-supplement mean	left temporal pole	0.123	0.316	0.517
BSL-supplement mean	left transverse temporal	0.076	0.537	0.702
BSL-supplement mean	right banks of the superior temporal sulcus	0.313	0.009	0.314
BSL-supplement mean	right caudal anterior cingulate	0.218	0.074	0.360
BSL-supplement mean	right caudal middle frontal	0.135	0.272	0.513
BSL-supplement mean	right cuneus	0.087	0.479	0.641
BSL-supplement mean	right entorhinal	-0.166	0.177	0.390
BSL-supplement mean	right frontal pole	0.013	0.918	0.969
BSL-supplement mean	right fusiform	0.230	0.059	0.360

BSL-supplement mean	right inferior parietal	0.221	0.069	0.360
BSL-supplement mean	right inferior temporal	0.261	0.032	0.314
BSL-supplement mean	right insula	0.204	0.095	0.378
BSL-supplement mean	right isthmus cingulate	-0.042	0.736	0.835
BSL-supplement mean	right lateral occipital	0.119	0.335	0.518
BSL-supplement mean	right lateral orbitofrontal	0.194	0.114	0.390
BSL-supplement mean	right lingual	0.047	0.705	0.824
BSL-supplement mean	right medial orbitofrontal	0.187	0.127	0.390
BSL-supplement mean	right middle temporal	0.167	0.173	0.390
BSL-supplement mean	right paracentral	-0.126	0.305	0.517
BSL-supplement mean	right parahippocampal	-0.068	0.583	0.735
BSL-supplement mean	right pars opercularis	0.223	0.067	0.360
BSL-supplement mean	right pars orbitalis	0.180	0.143	0.390
BSL-supplement mean	right pars triangularis	0.207	0.091	0.378
BSL-supplement mean	right pericalcarine	0.122	0.323	0.517
BSL-supplement mean	right postcentral	-0.011	0.926	0.969
BSL-supplement mean	right posterior cingulate	-0.001	0.996	0.996
BSL-supplement mean	right precentral	-0.030	0.810	0.903
BSL-supplement mean	right precuneus	0.191	0.119	0.390
BSL-supplement mean	right rostral anterior cingulate	0.233	0.056	0.360
BSL-supplement mean	right rostral middle frontal	0.188	0.124	0.390
BSL-supplement mean	right superior frontal	0.109	0.378	0.546
BSL-supplement mean	right superior parietal	0.100	0.415	0.588
BSL-supplement mean	right superior temporal	0.251	0.039	0.314
BSL-supplement mean	right supramarginal	0.087	0.481	0.641
BSL-supplement mean	right temporal pole	0.264	0.030	0.314
BSL-supplement mean	right transverse temporal	0.276	0.023	0.314
IPDE borderline	left banks of the superior temporal sulcus	0.270	0.026	0.344

IPDE borderline	left caudal anterior cingulate	0.015	0.901	0.958
IPDE borderline	left caudal middle frontal	0.126	0.306	0.535
IPDE borderline	left cuneus	0.216	0.076	0.415
IPDE borderline	left entorhinal	-0.034	0.783	0.887
IPDE borderline	left frontal pole	0.133	0.281	0.531
IPDE borderline	left fusiform	0.175	0.153	0.441
IPDE borderline	left inferior parietal	0.134	0.276	0.531
IPDE borderline	left inferior temporal	0.174	0.155	0.441
IPDE borderline	left insula	0.152	0.217	0.481
IPDE borderline	left isthmus cingulate	0.123	0.318	0.535
IPDE borderline	left lateral occipital	-0.124	0.315	0.535
IPDE borderline	left lateral orbitofrontal	0.123	0.319	0.535
IPDE borderline	left lingual	0.010	0.933	0.963
IPDE borderline	left medial orbitofrontal	0.042	0.736	0.863
IPDE borderline	left middle temporal	0.234	0.055	0.415
IPDE borderline	left paracentral	-0.022	0.858	0.941
IPDE borderline	left parahippocampal	-0.004	0.974	0.974
IPDE borderline	left pars opercularis	0.225	0.064	0.415
IPDE borderline	left pars orbitalis	0.086	0.484	0.686
IPDE borderline	left pars triangularis	0.195	0.111	0.420
IPDE borderline	left pericalcarine	0.028	0.821	0.915
IPDE borderline	left postcentral	0.174	0.156	0.441
IPDE borderline	left posterior cingulate	0.070	0.568	0.773
IPDE borderline	left precentral	0.093	0.451	0.667
IPDE borderline	left precuneus	0.116	0.346	0.559
IPDE borderline	left rostral anterior cingulate	0.018	0.886	0.957
IPDE borderline	left rostral middle frontal	0.187	0.126	0.428
IPDE borderline	left superior frontal	0.087	0.479	0.686

IPDE borderline	left superior parietal	0.055	0.654	0.828
IPDE borderline	left superior temporal	0.256	0.035	0.344
IPDE borderline	left supramarginal	0.181	0.139	0.441
IPDE borderline	left temporal pole	0.007	0.956	0.970
IPDE borderline	left transverse temporal	0.146	0.235	0.497
IPDE borderline	right banks of the superior temporal sulcus	0.110	0.372	0.588
IPDE borderline	right caudal anterior cingulate	0.152	0.215	0.481
IPDE borderline	right caudal middle frontal	0.151	0.218	0.481
IPDE borderline	right cuneus	0.010	0.935	0.963
IPDE borderline	right entorhinal	0.050	0.686	0.828
IPDE borderline	right frontal pole	0.151	0.219	0.481
IPDE borderline	right fusiform	0.188	0.125	0.428
IPDE borderline	right inferior parietal	0.159	0.194	0.481
IPDE borderline	right inferior temporal	0.248	0.041	0.350
IPDE borderline	right insula	0.293	0.015	0.344
IPDE borderline	right isthmus cingulate	-0.053	0.666	0.828
IPDE borderline	right lateral occipital	0.049	0.690	0.828
IPDE borderline	right lateral orbitofrontal	0.151	0.218	0.481
IPDE borderline	right lingual	0.038	0.759	0.874
IPDE borderline	right medial orbitofrontal	0.211	0.085	0.415
IPDE borderline	right middle temporal	0.206	0.092	0.415
IPDE borderline	right paracentral	-0.122	0.322	0.535
IPDE borderline	right parahippocampal	-0.049	0.694	0.828
IPDE borderline	right pars opercularis	0.108	0.382	0.590
IPDE borderline	right pars orbitalis	0.196	0.109	0.420
IPDE borderline	right pars triangularis	0.144	0.241	0.497
IPDE borderline	right pericalcarine	0.082	0.506	0.702
IPDE borderline	right postcentral	0.166	0.176	0.477

IPDE borderline	right posterior cingulate	0.141	0.250	0.501
IPDE borderline	right precentral	0.059	0.631	0.825
IPDE borderline	right precuneus	0.064	0.603	0.804
IPDE borderline	right rostral anterior cingulate	0.284	0.019	0.344
IPDE borderline	right rostral middle frontal	0.209	0.087	0.415
IPDE borderline	right superior frontal	0.202	0.099	0.420
IPDE borderline	right superior parietal	0.101	0.411	0.622
IPDE borderline	right superior temporal	0.261	0.032	0.344
IPDE borderline	right supramarginal	0.216	0.077	0.415
IPDE borderline	right temporal pole	0.285	0.018	0.344
IPDE borderline	right transverse temporal	0.256	0.035	0.344
IPO mean	left banks of the superior temporal sulcus	0.259	0.034	0.129
IPO mean	left caudal anterior cingulate	0.013	0.917	0.944
IPO mean	left caudal middle frontal	0.293	0.016	0.104
IPO mean	left cuneus	0.263	0.031	0.129
IPO mean	left entorhinal	0.061	0.625	0.726
IPO mean	left frontal pole	0.043	0.727	0.797
IPO mean	left fusiform	0.279	0.022	0.127
IPO mean	left inferior parietal	0.197	0.111	0.215
IPO mean	left inferior temporal	0.235	0.055	0.163
IPO mean	left insula	0.202	0.101	0.215
IPO mean	left isthmus cingulate	0.154	0.213	0.326
IPO mean	left lateral occipital	-0.028	0.820	0.872
IPO mean	left lateral orbitofrontal	0.173	0.160	0.260
IPO mean	left lingual	0.123	0.321	0.455
IPO mean	left medial orbitofrontal	0.146	0.239	0.353
IPO mean	left middle temporal	0.332	0.006	0.096
IPO mean	left paracentral	0.060	0.630	0.726

IPO mean	left parahippocampal	0.176	0.154	0.256
IPO mean	left pars opercularis	0.264	0.031	0.129
IPO mean	left pars orbitalis	0.110	0.377	0.503
IPO mean	left pars triangularis	0.296	0.015	0.104
IPO mean	left pericalcarine	0.068	0.582	0.695
IPO mean	left postcentral	0.183	0.138	0.235
IPO mean	left posterior cingulate	0.185	0.135	0.235
IPO mean	left precentral	0.153	0.216	0.326
IPO mean	left precuneus	0.201	0.102	0.215
IPO mean	left rostral anterior cingulate	0.114	0.358	0.488
IPO mean	left rostral middle frontal	0.246	0.045	0.160
IPO mean	left superior frontal	0.203	0.100	0.215
IPO mean	left superior parietal	0.101	0.418	0.536
IPO mean	left superior temporal	0.298	0.014	0.104
IPO mean	left supramarginal	0.260	0.034	0.129
IPO mean	left temporal pole	0.198	0.108	0.215
IPO mean	left transverse temporal	0.171	0.165	0.262
IPO mean	right banks of the superior temporal sulcus	0.144	0.246	0.357
IPO mean	right caudal anterior cingulate	0.355	0.003	0.073
IPO mean	right caudal middle frontal	0.291	0.017	0.104
IPO mean	right cuneus	0.114	0.359	0.488
IPO mean	right entorhinal	-0.083	0.505	0.624
IPO mean	right frontal pole	0.071	0.565	0.687
IPO mean	right fusiform	0.190	0.123	0.220
IPO mean	right inferior parietal	0.264	0.031	0.129
IPO mean	right inferior temporal	0.310	0.011	0.104
IPO mean	right insula	0.197	0.111	0.215
IPO mean	right isthmus cingulate	-0.035	0.776	0.838

IPO mean	right lateral occipital	0.058	0.643	0.729
IPO mean	right lateral orbitofrontal	0.190	0.123	0.220
IPO mean	right lingual	0.000	0.998	0.998
IPO mean	right medial orbitofrontal	0.241	0.049	0.160
IPO mean	right middle temporal	0.233	0.057	0.163
IPO mean	right paracentral	0.021	0.869	0.909
IPO mean	right parahippocampal	0.010	0.936	0.950
IPO mean	right pars opercularis	0.227	0.065	0.176
IPO mean	right pars orbitalis	0.215	0.080	0.202
IPO mean	right pars triangularis	0.205	0.096	0.215
IPO mean	right pericalcarine	0.048	0.702	0.782
IPO mean	right postcentral	0.105	0.397	0.519
IPO mean	right posterior cingulate	0.216	0.079	0.202
IPO mean	right precentral	0.090	0.467	0.588
IPO mean	right precuneus	0.243	0.048	0.160
IPO mean	right rostral anterior cingulate	0.397	<0.001	0.030
IPO mean	right rostral middle frontal	0.297	0.015	0.104
IPO mean	right superior frontal	0.237	0.053	0.163
IPO mean	right superior parietal	0.192	0.120	0.220
IPO mean	right superior temporal	0.259	0.034	0.129
IPO mean	right supramarginal	0.200	0.105	0.215
IPO mean	right temporal pole	0.413	<0.001	0.030
IPO mean	right transverse temporal	0.326	0.007	0.096
DERS total	left banks of the superior temporal sulcus	0.135	0.287	0.456
DERS total	left caudal anterior cingulate	0.071	0.575	0.686
DERS total	left caudal middle frontal	0.195	0.123	0.278
DERS total	left cuneus	0.146	0.250	0.435

DERS total	left entorhinal	0.082	0.521	0.651
DERS total	left frontal pole	0.103	0.419	0.559
DERS total	left fusiform	0.328	0.008	0.208
DERS total	left inferior parietal	0.242	0.054	0.215
DERS total	left inferior temporal	0.279	0.026	0.208
DERS total	left insula	0.118	0.353	0.490
DERS total	left isthmus cingulate	0.142	0.262	0.435
DERS total	left lateral occipital	0.040	0.751	0.785
DERS total	left lateral orbitofrontal	0.231	0.066	0.225
DERS total	left lingual	0.197	0.119	0.278
DERS total	left medial orbitofrontal	0.115	0.366	0.498
DERS total	left middle temporal	0.248	0.049	0.215
DERS total	left paracentral	-0.023	0.858	0.858
DERS total	left parahippocampal	0.061	0.630	0.714
DERS total	left pars opercularis	0.204	0.106	0.260
DERS total	left pars orbitalis	0.203	0.107	0.260
DERS total	left pars triangularis	0.260	0.038	0.213
DERS total	left pericalcarine	0.055	0.665	0.729
DERS total	left postcentral	0.171	0.177	0.364
DERS total	left posterior cingulate	0.124	0.328	0.484
DERS total	left precentral	0.129	0.310	0.479
DERS total	left precuneus	0.224	0.076	0.234
DERS total	left rostral anterior cingulate	0.148	0.242	0.435
DERS total	left rostral middle frontal	0.227	0.071	0.229
DERS total	left superior frontal	0.125	0.324	0.484
DERS total	left superior parietal	0.066	0.606	0.710
DERS total	left superior temporal	0.238	0.058	0.220
DERS total	left supramarginal	0.274	0.029	0.208

DERS total	left temporal pole	0.034	0.787	0.811
DERS total	left transverse temporal	0.246	0.050	0.215
DERS total	right banks of the superior temporal sulcus	0.057	0.654	0.729
DERS total	right caudal anterior cingulate	0.242	0.054	0.215
DERS total	right caudal middle frontal	0.151	0.233	0.435
DERS total	right cuneus	0.049	0.698	0.745
DERS total	right entorhinal	-0.063	0.622	0.714
DERS total	right frontal pole	0.156	0.219	0.435
DERS total	right fusiform	0.286	0.022	0.208
DERS total	right inferior parietal	0.301	0.016	0.208
DERS total	right inferior temporal	0.329	0.008	0.208
DERS total	right insula	0.272	0.029	0.208
DERS total	right isthmus cingulate	-0.085	0.506	0.649
DERS total	right lateral occipital	0.081	0.527	0.651
DERS total	right lateral orbitofrontal	0.270	0.031	0.208
DERS total	right lingual	0.123	0.335	0.484
DERS total	right medial orbitofrontal	0.246	0.050	0.215
DERS total	right middle temporal	0.266	0.034	0.210
DERS total	right paracentral	-0.032	0.800	0.812
DERS total	right parahippocampal	0.093	0.465	0.608
DERS total	right pars opercularis	0.152	0.232	0.435
DERS total	right pars orbitalis	0.235	0.062	0.221
DERS total	right pars triangularis	0.214	0.090	0.254
DERS total	right pericalcarine	0.135	0.288	0.456
DERS total	right postcentral	0.076	0.548	0.666
DERS total	right posterior cingulate	0.210	0.095	0.254
DERS total	right precentral	0.049	0.701	0.745
DERS total	right precuneus	0.210	0.096	0.254

DERS total	right rostral anterior cingulate	0.304	0.015	0.208
DERS total	right rostral middle frontal	0.286	0.022	0.208
DERS total	right superior frontal	0.143	0.259	0.435
DERS total	right superior parietal	0.119	0.351	0.490
DERS total	right superior temporal	0.185	0.142	0.302
DERS total	right supramarginal	0.188	0.138	0.302
DERS total	right temporal pole	0.209	0.097	0.254
DERS total	right transverse temporal	0.148	0.244	0.435
FDS20 mean	left banks of the superior temporal sulcus	0.287	0.018	0.104
FDS20 mean	left caudal anterior cingulate	0.100	0.415	0.463
FDS20 mean	left caudal middle frontal	0.316	0.009	0.089
FDS20 mean	left cuneus	0.213	0.082	0.150
FDS20 mean	left entorhinal	0.069	0.578	0.597
FDS20 mean	left frontal pole	0.205	0.094	0.164
FDS20 mean	left fusiform	0.314	0.009	0.089
FDS20 mean	left inferior parietal	0.261	0.032	0.104
FDS20 mean	left inferior temporal	0.280	0.021	0.104
FDS20 mean	left insula	0.067	0.588	0.597
FDS20 mean	left isthmus cingulate	0.177	0.150	0.203
FDS20 mean	left lateral occipital	0.230	0.059	0.122
FDS20 mean	left lateral orbitofrontal	0.257	0.034	0.104
FDS20 mean	left lingual	0.234	0.054	0.122
FDS20 mean	left medial orbitofrontal	0.259	0.033	0.104
FDS20 mean	left middle temporal	0.300	0.013	0.097
FDS20 mean	left paracentral	0.178	0.146	0.203
FDS20 mean	left parahippocampal	0.232	0.057	0.122
FDS20 mean	left pars opercularis	0.179	0.143	0.203
FDS20 mean	left pars orbitalis	0.271	0.025	0.104

FDS20 mean	left pars triangularis	0.285	0.018	0.104
FDS20 mean	left pericalcarine	0.216	0.077	0.146
FDS20 mean	left postcentral	0.139	0.260	0.315
FDS20 mean	left posterior cingulate	0.200	0.102	0.170
FDS20 mean	left precentral	0.181	0.139	0.203
FDS20 mean	left precuneus	0.264	0.030	0.104
FDS20 mean	left rostral anterior cingulate	0.116	0.346	0.405
FDS20 mean	left rostral middle frontal	0.254	0.037	0.104
FDS20 mean	left superior frontal	0.147	0.230	0.285
FDS20 mean	left superior parietal	0.111	0.369	0.425
FDS20 mean	left superior temporal	0.261	0.031	0.104
FDS20 mean	left supramarginal	0.252	0.038	0.104
FDS20 mean	left temporal pole	0.189	0.123	0.193
FDS20 mean	left transverse temporal	0.108	0.381	0.432
FDS20 mean	right banks of the superior temporal sulcus	0.190	0.121	0.193
FDS20 mean	right caudal anterior cingulate	0.305	0.011	0.097
FDS20 mean	right caudal middle frontal	0.188	0.125	0.193
FDS20 mean	right cuneus	0.167	0.173	0.231
FDS20 mean	right entorhinal	-0.044	0.722	0.722
FDS20 mean	right frontal pole	0.245	0.044	0.110
FDS20 mean	right fusiform	0.261	0.032	0.104
FDS20 mean	right inferior parietal	0.375	0.002	0.089
FDS20 mean	right inferior temporal	0.336	0.005	0.089
FDS20 mean	right insula	0.184	0.134	0.203
FDS20 mean	right isthmus cingulate	-0.095	0.443	0.486
FDS20 mean	right lateral occipital	0.272	0.025	0.104
FDS20 mean	right lateral orbitofrontal	0.314	0.009	0.089
FDS20 mean	right lingual	0.076	0.540	0.574

FDS20 mean	right medial orbitofrontal	0.248	0.041	0.108
FDS20 mean	right middle temporal	0.273	0.024	0.104
FDS20 mean	right paracentral	0.079	0.520	0.561
FDS20 mean	right parahippocampal	0.148	0.227	0.285
FDS20 mean	right pars opercularis	0.234	0.055	0.122
FDS20 mean	right pars orbitalis	0.253	0.037	0.104
FDS20 mean	right pars triangularis	0.202	0.098	0.166
FDS20 mean	right pericalcarine	0.258	0.034	0.104
FDS20 mean	right postcentral	0.068	0.579	0.597
FDS20 mean	right posterior cingulate	0.227	0.063	0.122
FDS20 mean	right precentral	0.148	0.229	0.285
FDS20 mean	right precuneus	0.327	0.007	0.089
FDS20 mean	right rostral anterior cingulate	0.323	0.007	0.089
FDS20 mean	right rostral middle frontal	0.228	0.061	0.122
FDS20 mean	right superior frontal	0.179	0.145	0.203
FDS20 mean	right superior parietal	0.205	0.093	0.164
FDS20 mean	right superior temporal	0.240	0.048	0.117
FDS20 mean	right supramarginal	0.153	0.212	0.278
FDS20 mean	right temporal pole	0.228	0.061	0.122
FDS20 mean	right transverse temporal	0.132	0.282	0.336
BDI-II sum	left banks of the superior temporal sulcus	0.233	0.055	0.236
BDI-II sum	left caudal anterior cingulate	0.074	0.548	0.653
BDI-II sum	left caudal middle frontal	0.191	0.119	0.247
BDI-II sum	left cuneus	0.068	0.579	0.679
BDI-II sum	left entorhinal	0.223	0.068	0.247
BDI-II sum	left frontal pole	-0.014	0.908	0.965
BDI-II sum	left fusiform	0.260	0.032	0.183
BDI-II sum	left inferior parietal	0.246	0.043	0.220

BDI-II sum	left inferior temporal	0.203	0.097	0.247
BDI-II sum	left insula	0.189	0.123	0.247
BDI-II sum	left isthmus cingulate	0.157	0.201	0.325
BDI-II sum	left lateral occipital	0.101	0.411	0.518
BDI-II sum	left lateral orbitofrontal	0.219	0.072	0.247
BDI-II sum	left lingual	0.151	0.220	0.348
BDI-II sum	left medial orbitofrontal	0.196	0.109	0.247
BDI-II sum	left middle temporal	0.233	0.055	0.236
BDI-II sum	left paracentral	0.101	0.411	0.518
BDI-II sum	left parahippocampal	0.102	0.410	0.518
BDI-II sum	left pars opercularis	0.110	0.373	0.498
BDI-II sum	left pars orbitalis	0.272	0.025	0.180
BDI-II sum	left pars triangularis	0.311	0.010	0.168
BDI-II sum	left pericalcarine	0.030	0.807	0.871
BDI-II sum	left postcentral	0.203	0.098	0.247
BDI-II sum	left posterior cingulate	0.051	0.682	0.786
BDI-II sum	left precentral	0.097	0.430	0.532
BDI-II sum	left precuneus	0.127	0.301	0.445
BDI-II sum	left rostral anterior cingulate	0.130	0.290	0.438
BDI-II sum	left rostral middle frontal	0.200	0.101	0.247
BDI-II sum	left superior frontal	0.140	0.256	0.396
BDI-II sum	left superior parietal	0.173	0.158	0.268
BDI-II sum	left superior temporal	0.272	0.025	0.180
BDI-II sum	left supramarginal	0.211	0.084	0.247
BDI-II sum	left temporal pole	0.120	0.328	0.448
BDI-II sum	left transverse temporal	0.265	0.029	0.180
BDI-II sum	right banks of the superior temporal sulcus	0.373	0.002	0.118
BDI-II sum	right caudal anterior cingulate	0.183	0.136	0.254

BDI-II sum	right caudal middle frontal	0.120	0.329	0.448
BDI-II sum	right cuneus	0.186	0.130	0.252
BDI-II sum	right entorhinal	-0.006	0.964	0.977
BDI-II sum	right frontal pole	0.123	0.318	0.448
BDI-II sum	right fusiform	0.175	0.153	0.266
BDI-II sum	right inferior parietal	0.316	0.009	0.168
BDI-II sum	right inferior temporal	0.166	0.177	0.293
BDI-II sum	right insula	0.197	0.107	0.247
BDI-II sum	right isthmus cingulate	0.004	0.977	0.977
BDI-II sum	right lateral occipital	0.007	0.956	0.977
BDI-II sum	right lateral orbitofrontal	0.191	0.119	0.247
BDI-II sum	right lingual	0.189	0.124	0.247
BDI-II sum	right medial orbitofrontal	0.244	0.045	0.220
BDI-II sum	right middle temporal	0.193	0.114	0.247
BDI-II sum	right paracentral	0.042	0.735	0.806
BDI-II sum	right parahippocampal	0.043	0.725	0.806
BDI-II sum	right pars opercularis	0.125	0.308	0.445
BDI-II sum	right pars orbitalis	0.316	0.009	0.168
BDI-II sum	right pars triangularis	0.206	0.092	0.247
BDI-II sum	right pericalcarine	0.198	0.105	0.247
BDI-II sum	right postcentral	0.196	0.110	0.247
BDI-II sum	right posterior cingulate	0.007	0.958	0.977
BDI-II sum	right precentral	0.085	0.489	0.594
BDI-II sum	right precuneus	0.212	0.083	0.247
BDI-II sum	right rostral anterior cingulate	0.285	0.019	0.180
BDI-II sum	right rostral middle frontal	0.295	0.015	0.180
BDI-II sum	right superior frontal	0.180	0.142	0.254
BDI-II sum	right superior parietal	0.180	0.142	0.254

BDI-II sum	right superior temporal	0.268	0.027	0.180
BDI-II sum	right supramarginal	0.191	0.119	0.247
BDI-II sum	right temporal pole	0.043	0.725	0.806
BDI-II sum	right transverse temporal	0.287	0.018	0.180

Supplementary Table S6. Correlations of psychometric findings and cortical thickness of patients with borderline personality disorder. Abbreviations: *BDI, Beck's depression inventory; BSL, borderline symptom list; DERS, difficulties in emotion regulation scale; FDS, Freiburger Dissoziationsskala; IPDE, International Personality Disorder Examination; IPO, Inventory of Personality Organization.*

Questionnaire	Brain Region	r	p-value	Adjusted p-value
BSL23 mean	Left Lateral Ventricle	0.039	0.754	0.928
BSL23 mean	Left Thalamus	0.012	0.920	0.945
BSL23 mean	Left Caudate	0.224	0.066	0.530
BSL23 mean	Left Putamen	-0.011	0.929	0.945
BSL23 mean	Left Pallidum	0.082	0.508	0.815
BSL23 mean	Left Hippocampus	0.134	0.275	0.815
BSL23 mean	Left Amygdala	0.075	0.541	0.815
BSL23 mean	Left Accumbens area	-0.008	0.945	0.945
BSL23 mean	Right Lateral Ventricle	0.111	0.366	0.815
BSL23 mean	Right Thalamus	-0.061	0.622	0.830
BSL23 mean	Right Caudate	0.253	0.037	0.530
BSL23 mean	Right Putamen	0.137	0.264	0.815
BSL23 mean	Right Pallidum	0.157	0.201	0.815
BSL23 mean	Right Hippocampus	0.094	0.446	0.815
BSL23 mean	Right Amygdala	0.072	0.560	0.815
BSL23 mean	Right Accumbens area	-0.088	0.476	0.815
BSL-supplement mean	Left Lateral Ventricle	0.150	0.222	0.510
BSL-supplement mean	Left Thalamus	0.069	0.574	0.731
BSL-supplement mean	Left Caudate	0.184	0.134	0.510
BSL-supplement mean	Left Putamen	0.084	0.494	0.731
BSL-supplement mean	Left Pallidum	0.114	0.355	0.632
BSL-supplement mean	Left Hippocampus	0.171	0.163	0.510
BSL-supplement mean	Left Amygdala	-0.067	0.585	0.731
BSL-supplement mean	Left Accumbens area	0.041	0.737	0.842
BSL-supplement mean	Right Lateral Ventricle	0.162	0.188	0.510
BSL-supplement mean	Right Thalamus	-0.013	0.919	0.958
BSL-supplement mean	Right Caudate	0.191	0.118	0.510

BSL-supplement mean	Right Putamen	0.191	0.119	0.510
BSL-supplement mean	Right Pallidum	0.124	0.313	0.625
BSL-supplement mean	Right Hippocampus	0.066	0.594	0.731
BSL-supplement mean	Right Amygdala	0.007	0.958	0.958
BSL-supplement mean	Right Accumbens area	-0.150	0.223	0.510
IPDE borderline	Left Lateral Ventricle	-0.085	0.490	0.944
IPDE borderline	Left Thalamus	0.121	0.325	0.944
IPDE borderline	Left Caudate	0.149	0.224	0.944
IPDE borderline	Left Putamen	0.005	0.969	0.969
IPDE borderline	Left Pallidum	0.015	0.903	0.963
IPDE borderline	Left Hippocampus	0.043	0.728	0.944
IPDE borderline	Left Amygdala	0.082	0.506	0.944
IPDE borderline	Left Accumbens area	-0.023	0.851	0.963
IPDE borderline	Right Lateral Ventricle	-0.037	0.767	0.944
IPDE borderline	Right Thalamus	0.191	0.118	0.944
IPDE borderline	Right Caudate	0.178	0.146	0.944
IPDE borderline	Right Putamen	0.045	0.714	0.944
IPDE borderline	Right Pallidum	0.075	0.542	0.944
IPDE borderline	Right Hippocampus	0.052	0.675	0.944
IPDE borderline	Right Amygdala	0.087	0.481	0.944
IPDE borderline	Right Accumbens area	0.078	0.528	0.944
IPO mean	Left Lateral Ventricle	-0.066	0.598	0.922
IPO mean	Left Thalamus	0.096	0.438	0.876
IPO mean	Left Caudate	0.104	0.402	0.876
IPO mean	Left Putamen	0.082	0.510	0.907
IPO mean	Left Pallidum	0.038	0.759	0.922
IPO mean	Left Hippocampus	0.041	0.742	0.922
IPO mean	Left Amygdala	0.012	0.922	0.922
IPO mean	Left Accumbens area	0.103	0.408	0.876
IPO mean	Right Lateral Ventricle	0.043	0.731	0.922

IPO mean	Right Thalamus	0.097	0.434	0.876
IPO mean	Right Caudate	0.149	0.229	0.876
IPO mean	Right Putamen	0.239	0.052	0.826
IPO mean	Right Pallidum	0.150	0.227	0.876
IPO mean	Right Hippocampus	-0.016	0.898	0.922
IPO mean	Right Amygdala	0.118	0.341	0.876
IPO mean	Right Accumbens area	0.019	0.877	0.922
DERS total	Left Lateral Ventricle	-0.009	0.945	0.945
DERS total	Left Thalamus	0.120	0.343	0.945
DERS total	Left Caudate	0.130	0.306	0.945
DERS total	Left Putamen	-0.103	0.416	0.945
DERS total	Left Pallidum	-0.048	0.704	0.945
DERS total	Left Hippocampus	0.086	0.499	0.945
DERS total	Left Amygdala	-0.045	0.725	0.945
DERS total	Left Accumbens area	-0.011	0.933	0.945
DERS total	Right Lateral Ventricle	-0.011	0.930	0.945
DERS total	Right Thalamus	0.036	0.777	0.945
DERS total	Right Caudate	0.135	0.287	0.945
DERS total	Right Putamen	-0.010	0.936	0.945
DERS total	Right Pallidum	0.058	0.646	0.945
DERS total	Right Hippocampus	0.018	0.885	0.945
DERS total	Right Amygdala	-0.105	0.410	0.945
DERS total	Right Accumbens area	-0.091	0.473	0.945
FDS20 mean	Left Lateral Ventricle	0.171	0.164	0.525
FDS20 mean	Left Thalamus	-0.084	0.498	0.735
FDS20 mean	Left Caudate	0.077	0.533	0.735
FDS20 mean	Left Putamen	0.070	0.570	0.735
FDS20 mean	Left Pallidum	0.039	0.754	0.754
FDS20 mean	Left Hippocampus	0.111	0.369	0.735
FDS20 mean	Left Amygdala	0.091	0.460	0.735

FDS20 mean	Left Accumbens area	0.050	0.688	0.735
FDS20 mean	Right Lateral Ventricle	0.222	0.068	0.402
FDS20 mean	Right Thalamus	-0.060	0.630	0.735
FDS20 mean	Right Caudate	0.078	0.526	0.735
FDS20 mean	Right Putamen	0.217	0.075	0.402
FDS20 mean	Right Pallidum	0.083	0.500	0.735
FDS20 mean	Right Hippocampus	0.186	0.129	0.517
FDS20 mean	Right Amygdala	0.221	0.070	0.402
FDS20 mean	Right Accumbens area	-0.049	0.689	0.735
BDI-II sum	Left Lateral Ventricle	0.116	0.348	0.517
BDI-II sum	Left Thalamus	0.158	0.198	0.453
BDI-II sum	Left Caudate	0.189	0.123	0.453
BDI-II sum	Left Putamen	-0.013	0.918	0.918
BDI-II sum	Left Pallidum	0.114	0.353	0.517
BDI-II sum	Left Hippocampus	0.176	0.151	0.453
BDI-II sum	Left Amygdala	0.109	0.378	0.517
BDI-II sum	Left Accumbens area	0.099	0.420	0.517
BDI-II sum	Right Lateral Ventricle	0.190	0.121	0.453
BDI-II sum	Right Thalamus	0.102	0.409	0.517
BDI-II sum	Right Caudate	0.169	0.168	0.453
BDI-II sum	Right Putamen	0.113	0.360	0.517
BDI-II sum	Right Pallidum	0.164	0.180	0.453
BDI-II sum	Right Hippocampus	0.068	0.581	0.664
BDI-II sum	Right Amygdala	0.226	0.064	0.453
BDI-II sum	Right Accumbens area	0.022	0.860	0.917

Supplementary Table S7. Correlations of psychometric findings and subcortical volumes of patients with borderline personality disorder. Abbreviations: *BDI, Beck's depression inventory; BSL, borderline symptom list; DERS, difficulties in emotion regulation scale; FDS, Freiburger Dissoziationsskala; IPDE, International Personality Disorder Examination; IPO, Inventory of Personality Organization*

Questionnaire	Brain Region	r	p-value	adjusted p-value
BSL23 mean	GM/TIV corrected	-0.088	0.476	0.891
BSL23 mean	WM/TIV corrected	0.017	0.891	0.891
BSL23 mean	CSF/TIV corrected	0.059	0.634	0.891
BSL23 mean	TIV corrected	-0.018	0.883	0.891
BSL23 mean	Cerebellum/TIV corrected	0.022	0.860	0.891
BSL-supplement mean	GM/TIV corrected	-0.019	0.876	0.946
BSL-supplement mean	WM/TIV corrected	0.052	0.671	0.946
BSL-supplement mean	CSF/TIV corrected	0.008	0.946	0.946
BSL-supplement mean	TIV corrected	0.101	0.411	0.946
BSL-supplement mean	Cerebellum/TIV corrected	-0.081	0.512	0.946
IPDE borderline	GM/TIV corrected	-0.029	0.812	0.956
IPDE borderline	WM/TIV corrected	-0.065	0.601	0.956
IPDE borderline	CSF/TIV corrected	0.049	0.691	0.956
IPDE borderline	TIV corrected	0.110	0.370	0.956
IPDE borderline	Cerebellum/TIV corrected	-0.007	0.956	0.956
IPO mean	GM/TIV corrected	0.128	0.303	0.873
IPO mean	WM/TIV corrected	-0.068	0.586	0.873
IPO mean	CSF/TIV corrected	-0.034	0.785	0.873
IPO mean	TIV corrected	-0.044	0.722	0.873
IPO mean	Cerebellum/TIV corrected	0.020	0.873	0.873
DERS total	GM/TIV corrected	0.057	0.653	0.653
DERS total	WM/TIV corrected	-0.166	0.191	0.653
DERS total	CSF/TIV corrected	0.076	0.552	0.653
DERS total	TIV corrected	-0.059	0.642	0.653
DERS total	Cerebellum/TIV corrected	0.074	0.563	0.653
FDS20 mean	GM/TIV corrected	0.042	0.734	0.874
FDS20 mean	WM/TIV corrected	-0.122	0.322	0.874
FDS20 mean	CSF/TIV corrected	0.063	0.611	0.874
FDS20 mean	TIV corrected	0.026	0.831	0.874
FDS20 mean	Cerebellum/TIV corrected	0.020	0.874	0.874
BDI-II sum	GM/TIV corrected	0.134	0.274	0.274
BDI-II sum	WM/TIV corrected	0.152	0.216	0.274
BDI-II sum	CSF/TIV corrected	-0.174	0.156	0.274

BDI-II sum	TIV corrected	-0.232	0.057	0.274
BDI-II sum	Cerebellum/TIV corrected	0.135	0.272	0.274

Supplementary Table S8. Correlations of psychometric findings and global volume measures of patients with borderline personality disorder. Abbreviations: *BDI, Beck's depression inventory; BSL, borderline symptom list; CSF, cerebrospinal fluid; DERS, difficulties in emotion regulation scale; FDS, Freiburger Dissoziationsskala; GM, grey matter; IPDE, International Personality Disorder Examination; IPO, Inventory of Personality Organization; TIV, total intracranial volume; WM, white matter.*

Neuropsychological Test	Brain Region	r	p-value	adjusted p-value
IQ CFT20R	left banks of the superior temporal sulcus	0.105	0.425	0.994
IQ CFT20R	left caudal anterior cingulate	0.160	0.222	0.994
IQ CFT20R	left caudal middle frontal	0.083	0.530	0.994
IQ CFT20R	left cuneus	0.054	0.681	0.994
IQ CFT20R	left entorhinal	0.091	0.492	0.994
IQ CFT20R	left frontal pole	-0.058	0.657	0.994
IQ CFT20R	left fusiform	0.063	0.630	0.994
IQ CFT20R	left inferior parietal	-0.010	0.942	0.994
IQ CFT20R	left inferior temporal	-0.042	0.748	0.994
IQ CFT20R	left insula	0.103	0.434	0.994
IQ CFT20R	left isthmus cingulate	-0.049	0.709	0.994
IQ CFT20R	left lateral occipital	0.205	0.116	0.994
IQ CFT20R	left lateral orbitofrontal	-0.059	0.654	0.994
IQ CFT20R	left lingual	-0.002	0.989	0.994
IQ CFT20R	left medial orbitofrontal	0.044	0.736	0.994
IQ CFT20R	left middle temporal	0.121	0.357	0.994
IQ CFT20R	left paracentral	-0.086	0.515	0.994
IQ CFT20R	left parahippocampal	0.143	0.275	0.994
IQ CFT20R	left pars opercularis	0.104	0.427	0.994
IQ CFT20R	left pars orbitalis	-0.020	0.882	0.994
IQ CFT20R	left pars triangularis	0.039	0.769	0.994
IQ CFT20R	left pericalcarine	-0.098	0.457	0.994
IQ CFT20R	left postcentral	-0.074	0.576	0.994
IQ CFT20R	left posterior cingulate	0.156	0.234	0.994
IQ CFT20R	left precentral	0.082	0.534	0.994
IQ CFT20R	left precuneus	0.048	0.716	0.994
IQ CFT20R	left rostral anterior cingulate	0.025	0.851	0.994

IQ CFT20R	left rostral middle frontal	-0.139	0.288	0.994
IQ CFT20R	left superior frontal	-0.008	0.950	0.994
IQ CFT20R	left superior parietal	-0.063	0.630	0.994
IQ CFT20R	left superior temporal	0.141	0.284	0.994
IQ CFT20R	left supramarginal	-0.023	0.860	0.994
IQ CFT20R	left temporal pole	-0.051	0.700	0.994
IQ CFT20R	left transverse temporal	-0.016	0.901	0.994
IQ CFT20R	right banks of the superior temporal sulcus	0.005	0.968	0.994
IQ CFT20R	right caudal anterior cingulate	0.103	0.434	0.994
IQ CFT20R	right caudal middle frontal	0.086	0.512	0.994
IQ CFT20R	right cuneus	0.017	0.899	0.994
IQ CFT20R	right entorhinal	-0.030	0.818	0.994
IQ CFT20R	right frontal pole	0.014	0.917	0.994
IQ CFT20R	right fusiform	0.006	0.964	0.994
IQ CFT20R	right inferior parietal	0.083	0.529	0.994
IQ CFT20R	right inferior temporal	0.097	0.460	0.994
IQ CFT20R	right insula	0.200	0.125	0.994
IQ CFT20R	right isthmus cingulate	-0.026	0.842	0.994
IQ CFT20R	right lateral occipital	-0.001	0.994	0.994
IQ CFT20R	right lateral orbitofrontal	-0.078	0.555	0.994
IQ CFT20R	right lingual	-0.099	0.452	0.994
IQ CFT20R	right medial orbitofrontal	0.019	0.888	0.994
IQ CFT20R	right middle temporal	0.048	0.714	0.994
IQ CFT20R	right paracentral	-0.060	0.650	0.994
IQ CFT20R	right parahippocampal	-0.032	0.806	0.994
IQ CFT20R	right pars opercularis	0.100	0.447	0.994
IQ CFT20R	right pars orbitalis	0.018	0.893	0.994
IQ CFT20R	right pars triangularis	0.056	0.673	0.994
IQ CFT20R	right pericalcarine	-0.094	0.474	0.994

IQ CFT20R	right postcentral	-0.079	0.550	0.994
IQ CFT20R	right posterior cingulate	0.070	0.596	0.994
IQ CFT20R	right precentral	0.046	0.727	0.994
IQ CFT20R	right precuneus	0.112	0.395	0.994
IQ CFT20R	right rostral anterior cingulate	0.021	0.873	0.994
IQ CFT20R	right rostral middle frontal	0.003	0.984	0.994
IQ CFT20R	right superior frontal	-0.071	0.592	0.994
IQ CFT20R	right superior parietal	-0.036	0.784	0.994
IQ CFT20R	right superior temporal	0.069	0.602	0.994
IQ CFT20R	right supramarginal	0.023	0.860	0.994
IQ CFT20R	right temporal pole	-0.065	0.624	0.994
IQ CFT20R	right transverse temporal	-0.109	0.406	0.994
VLMT Learning	left banks of the superior temporal sulcus	0.006	0.961	0.997
VLMT Learning	left caudal anterior cingulate	0.047	0.704	0.997
VLMT Learning	left caudal middle frontal	0.056	0.649	0.997
VLMT Learning	left cuneus	0.040	0.743	0.997
VLMT Learning	left entorhinal	-0.260	0.031	0.997
VLMT Learning	left frontal pole	-0.123	0.315	0.997
VLMT Learning	left fusiform	-0.094	0.441	0.997
VLMT Learning	left inferior parietal	0.002	0.987	0.997
VLMT Learning	left inferior temporal	-0.064	0.602	0.997
VLMT Learning	left insula	-0.067	0.584	0.997
VLMT Learning	left isthmus cingulate	0.090	0.461	0.997
VLMT Learning	left lateral occipital	0.077	0.527	0.997
VLMT Learning	left lateral orbitofrontal	-0.047	0.703	0.997
VLMT Learning	left lingual	0.008	0.948	0.997
VLMT Learning	left medial orbitofrontal	0.096	0.431	0.997
VLMT Learning	left middle temporal	-0.063	0.606	0.997
VLMT Learning	left paracentral	0.015	0.900	0.997
VLMT Learning	left parahippocampal	-0.055	0.656	0.997

VLMT Learning	left pars opercularis	-0.023	0.848	0.997
VLMT Learning	left pars orbitalis	0.050	0.683	0.997
VLMT Learning	left pars triangularis	-0.046	0.709	0.997
VLMT Learning	left pericalcarine	-0.108	0.375	0.997
VLMT Learning	left postcentral	-0.016	0.896	0.997
VLMT Learning	left posterior cingulate	-0.015	0.904	0.997
VLMT Learning	left precentral	-0.036	0.769	0.997
VLMT Learning	left precuneus	0.024	0.846	0.997
VLMT Learning	left rostral anterior cingulate	-0.017	0.887	0.997
VLMT Learning	left rostral middle frontal	0.017	0.889	0.997
VLMT Learning	left superior frontal	0.009	0.944	0.997
VLMT Learning	left superior parietal	-0.085	0.489	0.997
VLMT Learning	left superior temporal	-0.110	0.368	0.997
VLMT Learning	left supramarginal	-0.020	0.869	0.997
VLMT Learning	left temporal pole	0.023	0.848	0.997
VLMT Learning	left transverse temporal	-0.056	0.647	0.997
VLMT Learning	right banks of the superior temporal sulcus	-0.000	0.997	0.997
VLMT Learning	right caudal anterior cingulate	-0.084	0.493	0.997
VLMT Learning	right caudal middle frontal	0.074	0.548	0.997
VLMT Learning	right cuneus	0.086	0.481	0.997
VLMT Learning	right entorhinal	-0.185	0.129	0.997
VLMT Learning	right frontal pole	0.001	0.996	0.997
VLMT Learning	right fusiform	-0.075	0.540	0.997
VLMT Learning	right inferior parietal	-0.019	0.880	0.997
VLMT Learning	right inferior temporal	-0.059	0.630	0.997
VLMT Learning	right insula	0.095	0.438	0.997
VLMT Learning	right isthmus cingulate	-0.107	0.381	0.997
VLMT Learning	right lateral occipital	-0.106	0.384	0.997
VLMT Learning	right lateral orbitofrontal	0.033	0.787	0.997

VLMT Learning	right lingual	-0.019	0.878	0.997
VLMT Learning	right medial orbitofrontal	0.093	0.446	0.997
VLMT Learning	right middle temporal	0.007	0.956	0.997
VLMT Learning	right paracentral	0.066	0.590	0.997
VLMT Learning	right parahippocampal	0.049	0.688	0.997
VLMT Learning	right pars opercularis	-0.089	0.468	0.997
VLMT Learning	right pars orbitalis	0.027	0.825	0.997
VLMT Learning	right pars triangularis	-0.032	0.796	0.997
VLMT Learning	right pericalcarine	-0.058	0.638	0.997
VLMT Learning	right postcentral	-0.126	0.304	0.997
VLMT Learning	right posterior cingulate	0.005	0.969	0.997
VLMT Learning	right precentral	0.045	0.716	0.997
VLMT Learning	right precuneus	0.028	0.818	0.997
VLMT Learning	right rostral anterior cingulate	0.115	0.348	0.997
VLMT Learning	right rostral middle frontal	0.033	0.790	0.997
VLMT Learning	right superior frontal	-0.013	0.913	0.997
VLMT Learning	right superior parietal	-0.078	0.526	0.997
VLMT Learning	right superior temporal	-0.154	0.206	0.997
VLMT Learning	right supramarginal	-0.117	0.338	0.997
VLMT Learning	right temporal pole	0.002	0.985	0.997
VLMT Learning	right transverse temporal	0.052	0.670	0.997
VLMT Recognition	left banks of the superior temporal sulcus	-0.151	0.214	0.924
VLMT Recognition	left caudal anterior cingulate	-0.162	0.182	0.924
VLMT Recognition	left caudal middle frontal	-0.098	0.425	0.924
VLMT Recognition	left cuneus	0.023	0.850	0.924
VLMT Recognition	left entorhinal	0.192	0.113	0.924
VLMT Recognition	left frontal pole	-0.039	0.750	0.924
VLMT Recognition	left fusiform	-0.109	0.371	0.924
VLMT Recognition	left inferior parietal	-0.064	0.600	0.924
VLMT Recognition	left inferior temporal	-0.088	0.473	0.924

VLMT Recognition	left insula	-0.002	0.984	0.994
VLMT Recognition	left isthmus cingulate	-0.050	0.684	0.924
VLMT Recognition	left lateral occipital	-0.067	0.582	0.924
VLMT Recognition	left lateral orbitofrontal	-0.052	0.673	0.924
VLMT Recognition	left lingual	-0.142	0.244	0.924
VLMT Recognition	left medial orbitofrontal	-0.105	0.389	0.924
VLMT Recognition	left middle temporal	-0.089	0.469	0.924
VLMT Recognition	left paracentral	-0.131	0.285	0.924
VLMT Recognition	left parahippocampal	-0.249	0.039	0.881
VLMT Recognition	left pars opercularis	-0.035	0.773	0.924
VLMT Recognition	left pars orbitalis	-0.086	0.481	0.924
VLMT Recognition	left pars triangularis	-0.060	0.626	0.924
VLMT Recognition	left pericalcarine	-0.152	0.212	0.924
VLMT Recognition	left postcentral	-0.072	0.556	0.924
VLMT Recognition	left posterior cingulate	-0.065	0.597	0.924
VLMT Recognition	left precentral	-0.105	0.389	0.924
VLMT Recognition	left precuneus	-0.094	0.444	0.924
VLMT Recognition	left rostral anterior cingulate	-0.121	0.323	0.924
VLMT Recognition	left rostral middle frontal	-0.111	0.365	0.924
VLMT Recognition	left superior frontal	-0.034	0.781	0.924
VLMT Recognition	left superior parietal	-0.035	0.778	0.924
VLMT Recognition	left superior temporal	-0.104	0.397	0.924
VLMT Recognition	left supramarginal	-0.062	0.615	0.924
VLMT Recognition	left temporal pole	-0.178	0.143	0.924
VLMT Recognition	left transverse temporal	-0.087	0.476	0.924
VLMT Recognition	right banks of the superior temporal sulcus	-0.102	0.405	0.924
VLMT Recognition	right caudal anterior cingulate	-0.290	0.016	0.841
VLMT Recognition	right caudal middle frontal	-0.031	0.802	0.924
VLMT Recognition	right cuneus	0.065	0.594	0.924

VLMT Recognition	right entorhinal	0.039	0.747	0.924
VLMT Recognition	right frontal pole	-0.020	0.870	0.924
VLMT Recognition	right fusiform	0.038	0.755	0.924
VLMT Recognition	right inferior parietal	-0.106	0.388	0.924
VLMT Recognition	right inferior temporal	0.047	0.699	0.924
VLMT Recognition	right insula	-0.132	0.281	0.924
VLMT Recognition	right isthmus cingulate	0.067	0.585	0.924
VLMT Recognition	right lateral occipital	-0.047	0.701	0.924
VLMT Recognition	right lateral orbitofrontal	-0.108	0.376	0.924
VLMT Recognition	right lingual	-0.270	0.025	0.841
VLMT Recognition	right medial orbitofrontal	-0.198	0.102	0.924
VLMT Recognition	right middle temporal	-0.021	0.864	0.924
VLMT Recognition	right paracentral	-0.133	0.275	0.924
VLMT Recognition	right parahippocampal	-0.114	0.349	0.924
VLMT Recognition	right pars opercularis	0.022	0.855	0.924
VLMT Recognition	right pars orbitalis	-0.027	0.827	0.924
VLMT Recognition	right pars triangularis	0.089	0.466	0.924
VLMT Recognition	right pericalcarine	-0.090	0.464	0.924
VLMT Recognition	right postcentral	-0.001	0.994	0.994
VLMT Recognition	right posterior cingulate	-0.099	0.416	0.924
VLMT Recognition	right precentral	-0.063	0.606	0.924
VLMT Recognition	right precuneus	-0.107	0.383	0.924
VLMT Recognition	right rostral anterior cingulate	-0.022	0.856	0.924
VLMT Recognition	right rostral middle frontal	-0.014	0.906	0.948
VLMT Recognition	right superior frontal	-0.041	0.737	0.924
VLMT Recognition	right superior parietal	0.006	0.963	0.993
VLMT Recognition	right superior temporal	0.045	0.710	0.924
VLMT Recognition	right supramarginal	-0.023	0.853	0.924
VLMT Recognition	right temporal pole	-0.140	0.252	0.924
VLMT Recognition	right transverse temporal	-0.054	0.661	0.924

VLMT Consolidation	left banks of the superior temporal sulcus	-0.073	0.554	0.996
VLMT Consolidation	left caudal anterior cingulate	-0.182	0.135	0.996
VLMT Consolidation	left caudal middle frontal	-0.005	0.966	0.996
VLMT Consolidation	left cuneus	0.047	0.701	0.996
VLMT Consolidation	left entorhinal	0.059	0.629	0.996
VLMT Consolidation	left frontal pole	0.016	0.899	0.996
VLMT Consolidation	left fusiform	0.023	0.854	0.996
VLMT Consolidation	left inferior parietal	-0.107	0.381	0.996
VLMT Consolidation	left inferior temporal	0.109	0.373	0.996
VLMT Consolidation	left insula	0.174	0.153	0.996
VLMT Consolidation	left isthmus cingulate	-0.144	0.238	0.996
VLMT Consolidation	left lateral occipital	-0.102	0.404	0.996
VLMT Consolidation	left lateral orbitofrontal	0.140	0.252	0.996
VLMT Consolidation	left lingual	-0.104	0.394	0.996
VLMT Consolidation	left medial orbitofrontal	-0.098	0.424	0.996
VLMT Consolidation	left middle temporal	0.068	0.581	0.996
VLMT Consolidation	left paracentral	-0.128	0.294	0.996
VLMT Consolidation	left parahippocampal	-0.177	0.145	0.996
VLMT Consolidation	left pars opercularis	0.068	0.578	0.996
VLMT Consolidation	left pars orbitalis	0.034	0.783	0.996
VLMT Consolidation	left pars triangularis	0.064	0.604	0.996
VLMT Consolidation	left pericalcarine	0.011	0.926	0.996
VLMT Consolidation	left postcentral	-0.024	0.843	0.996
VLMT Consolidation	left posterior cingulate	-0.019	0.878	0.996
VLMT Consolidation	left precentral	-0.027	0.825	0.996
VLMT Consolidation	left precuneus	-0.019	0.875	0.996
VLMT Consolidation	left rostral anterior cingulate	0.000	1.000	1.000
VLMT Consolidation	left rostral middle frontal	0.040	0.744	0.996
VLMT Consolidation	left superior frontal	-0.010	0.938	0.996
VLMT Consolidation	left superior parietal	-0.065	0.596	0.996

VLMT Consolidation	left superior temporal	-0.019	0.877	0.996
VLMT Consolidation	left supramarginal	0.008	0.949	0.996
VLMT Consolidation	left temporal pole	-0.076	0.536	0.996
VLMT Consolidation	left transverse temporal	-0.061	0.621	0.996
VLMT Consolidation	right banks of the superior temporal sulcus	-0.101	0.409	0.996
VLMT Consolidation	right caudal anterior cingulate	-0.202	0.096	0.996
VLMT Consolidation	right caudal middle frontal	-0.009	0.942	0.996
VLMT Consolidation	right cuneus	0.161	0.188	0.996
VLMT Consolidation	right entorhinal	-0.008	0.951	0.996
VLMT Consolidation	right frontal pole	-0.009	0.940	0.996
VLMT Consolidation	right fusiform	0.152	0.211	0.996
VLMT Consolidation	right inferior parietal	-0.039	0.748	0.996
VLMT Consolidation	right inferior temporal	0.205	0.091	0.996
VLMT Consolidation	right insula	-0.072	0.555	0.996
VLMT Consolidation	right isthmus cingulate	-0.031	0.798	0.996
VLMT Consolidation	right lateral occipital	-0.062	0.616	0.996
VLMT Consolidation	right lateral orbitofrontal	0.005	0.967	0.996
VLMT Consolidation	right lingual	-0.217	0.073	0.996
VLMT Consolidation	right medial orbitofrontal	0.035	0.774	0.996
VLMT Consolidation	right middle temporal	0.096	0.432	0.996
VLMT Consolidation	right paracentral	-0.084	0.491	0.996
VLMT Consolidation	right parahippocampal	-0.036	0.771	0.996
VLMT Consolidation	right pars opercularis	0.062	0.614	0.996
VLMT Consolidation	right pars orbitalis	0.082	0.502	0.996
VLMT Consolidation	right pars triangularis	0.174	0.152	0.996
VLMT Consolidation	right pericalcarine	0.045	0.714	0.996
VLMT Consolidation	right postcentral	0.013	0.914	0.996
VLMT Consolidation	right posterior cingulate	0.016	0.895	0.996
VLMT Consolidation	right precentral	0.038	0.755	0.996

VLMT Consolidation	right precuneus	-0.012	0.922	0.996
VLMT Consolidation	right rostral anterior cingulate	-0.027	0.823	0.996
VLMT Consolidation	right rostral middle frontal	0.053	0.667	0.996
VLMT Consolidation	right superior frontal	-0.038	0.760	0.996
VLMT Consolidation	right superior parietal	0.000	1.000	1.000
VLMT Consolidation	right superior temporal	0.061	0.621	0.996
VLMT Consolidation	right supramarginal	0.047	0.701	0.996
VLMT Consolidation	right temporal pole	-0.025	0.838	0.996
VLMT Consolidation	right transverse temporal	-0.015	0.906	0.996
Alertness no warning tone	left banks of the superior temporal sulcus	-0.047	0.713	0.983
Alertness no warning tone	left caudal anterior cingulate	0.199	0.115	0.983
Alertness no warning tone	left caudal middle frontal	0.040	0.755	0.983
Alertness no warning tone	left cuneus	0.005	0.968	0.983
Alertness no warning tone	left entorhinal	-0.007	0.957	0.983
Alertness no warning tone	left frontal pole	0.127	0.318	0.983
Alertness no warning tone	left fusiform	-0.007	0.958	0.983
Alertness no warning tone	left inferior parietal	-0.069	0.590	0.983
Alertness no warning tone	left inferior temporal	0.102	0.422	0.983
Alertness no warning tone	left insula	0.104	0.413	0.983
Alertness no warning tone	left isthmus cingulate	0.025	0.843	0.983
Alertness no warning tone	left lateral occipital	-0.056	0.658	0.983
Alertness no warning tone	left lateral orbitofrontal	0.069	0.589	0.983
Alertness no warning tone	left lingual	-0.018	0.885	0.983
Alertness no warning tone	left medial orbitofrontal	-0.061	0.633	0.983
Alertness no warning tone	left middle temporal	-0.079	0.533	0.983
Alertness no warning tone	left paracentral	0.026	0.840	0.983
Alertness no warning tone	left parahippocampal	0.086	0.497	0.983
Alertness no warning tone	left pars opercularis	0.202	0.110	0.983
Alertness no warning tone	left pars orbitalis	0.066	0.602	0.983
Alertness no warning tone	left pars triangularis	0.147	0.245	0.983

Alertness no warning tone	left pericalcarine	-0.129	0.311	0.983
Alertness no warning tone	left postcentral	0.042	0.743	0.983
Alertness no warning tone	left posterior cingulate	0.136	0.283	0.983
Alertness no warning tone	left precentral	0.032	0.801	0.983
Alertness no warning tone	left precuneus	0.035	0.784	0.983
Alertness no warning tone	left rostral anterior cingulate	-0.047	0.710	0.983
Alertness no warning tone	left rostral middle frontal	-0.035	0.785	0.983
Alertness no warning tone	left superior frontal	0.008	0.951	0.983
Alertness no warning tone	left superior parietal	-0.019	0.880	0.983
Alertness no warning tone	left superior temporal	-0.017	0.892	0.983
Alertness no warning tone	left supramarginal	-0.014	0.915	0.983
Alertness no warning tone	left temporal pole	-0.104	0.415	0.983
Alertness no warning tone	left transverse temporal	0.063	0.619	0.983
Alertness no warning tone	right banks of the superior temporal sulcus	0.155	0.222	0.983
Alertness no warning tone	right caudal anterior cingulate	0.096	0.450	0.983
Alertness no warning tone	right caudal middle frontal	0.090	0.481	0.983
Alertness no warning tone	right cuneus	-0.148	0.242	0.983
Alertness no warning tone	right entorhinal	-0.057	0.657	0.983
Alertness no warning tone	right frontal pole	0.128	0.314	0.983
Alertness no warning tone	right fusiform	0.139	0.275	0.983
Alertness no warning tone	right inferior parietal	-0.008	0.947	0.983
Alertness no warning tone	right inferior temporal	-0.069	0.589	0.983
Alertness no warning tone	right insula	0.035	0.784	0.983
Alertness no warning tone	right isthmus cingulate	0.026	0.837	0.983
Alertness no warning tone	right lateral occipital	-0.038	0.764	0.983
Alertness no warning tone	right lateral orbitofrontal	0.095	0.457	0.983
Alertness no warning tone	right lingual	0.045	0.727	0.983
Alertness no warning tone	right medial orbitofrontal	0.037	0.770	0.983
Alertness no warning tone	right middle temporal	0.014	0.910	0.983

Alertness no warning tone	right paracentral	0.001	0.995	0.995
Alertness no warning tone	right parahippocampal	0.034	0.791	0.983
Alertness no warning tone	right pars opercularis	0.039	0.760	0.983
Alertness no warning tone	right pars orbitalis	-0.012	0.926	0.983
Alertness no warning tone	right pars triangularis	0.125	0.323	0.983
Alertness no warning tone	right pericalcarine	0.029	0.822	0.983
Alertness no warning tone	right postcentral	-0.058	0.652	0.983
Alertness no warning tone	right posterior cingulate	-0.121	0.341	0.983
Alertness no warning tone	right precentral	-0.030	0.814	0.983
Alertness no warning tone	right precuneus	0.018	0.889	0.983
Alertness no warning tone	right rostral anterior cingulate	0.026	0.840	0.983
Alertness no warning tone	right rostral middle frontal	0.084	0.510	0.983
Alertness no warning tone	right superior frontal	0.026	0.840	0.983
Alertness no warning tone	right superior parietal	0.067	0.601	0.983
Alertness no warning tone	right superior temporal	0.122	0.339	0.983
Alertness no warning tone	right supramarginal	-0.053	0.675	0.983
Alertness no warning tone	right temporal pole	0.158	0.212	0.983
Alertness no warning tone	right transverse temporal	0.095	0.453	0.983
Alertness warning tone	left banks of the superior temporal sulcus	-0.095	0.455	0.987
Alertness warning tone	left caudal anterior cingulate	0.211	0.094	0.987
Alertness warning tone	left caudal middle frontal	0.055	0.669	0.987
Alertness warning tone	left cuneus	-0.038	0.768	0.987
Alertness warning tone	left entorhinal	0.135	0.287	0.987
Alertness warning tone	left frontal pole	0.103	0.418	0.987
Alertness warning tone	left fusiform	-0.020	0.876	0.987
Alertness warning tone	left inferior parietal	-0.074	0.560	0.987
Alertness warning tone	left inferior temporal	0.074	0.559	0.987
Alertness warning tone	left insula	0.061	0.632	0.987
Alertness warning tone	left isthmus cingulate	-0.023	0.858	0.987
Alertness warning tone	left lateral occipital	-0.063	0.619	0.987

Alertness warning tone	left lateral orbitofrontal	0.018	0.886	0.987
Alertness warning tone	left lingual	-0.100	0.433	0.987
Alertness warning tone	left medial orbitofrontal	-0.096	0.449	0.987
Alertness warning tone	left middle temporal	-0.119	0.349	0.987
Alertness warning tone	left paracentral	0.050	0.697	0.987
Alertness warning tone	left parahippocampal	0.151	0.234	0.987
Alertness warning tone	left pars opercularis	0.234	0.063	0.987
Alertness warning tone	left pars orbitalis	0.080	0.532	0.987
Alertness warning tone	left pars triangularis	0.074	0.562	0.987
Alertness warning tone	left pericalcarine	-0.124	0.328	0.987
Alertness warning tone	left postcentral	0.046	0.718	0.987
Alertness warning tone	left posterior cingulate	0.067	0.599	0.987
Alertness warning tone	left precentral	0.120	0.343	0.987
Alertness warning tone	left precuneus	0.070	0.583	0.987
Alertness warning tone	left rostral anterior cingulate	-0.045	0.723	0.987
Alertness warning tone	left rostral middle frontal	-0.070	0.583	0.987
Alertness warning tone	left superior frontal	0.046	0.716	0.987
Alertness warning tone	left superior parietal	0.015	0.908	0.987
Alertness warning tone	left superior temporal	-0.092	0.467	0.987
Alertness warning tone	left supramarginal	-0.018	0.887	0.987
Alertness warning tone	left temporal pole	-0.027	0.829	0.987
Alertness warning tone	left transverse temporal	-0.038	0.767	0.987
Alertness warning tone	right banks of the superior temporal sulcus	0.051	0.689	0.987
Alertness warning tone	right caudal anterior cingulate	0.027	0.829	0.987
Alertness warning tone	right caudal middle frontal	0.133	0.296	0.987
Alertness warning tone	right cuneus	-0.223	0.077	0.987
Alertness warning tone	right entorhinal	0.006	0.964	0.987
Alertness warning tone	right frontal pole	0.116	0.361	0.987
Alertness warning tone	right fusiform	0.023	0.855	0.987

Alertness warning tone	right inferior parietal	-0.049	0.699	0.987
Alertness warning tone	right inferior temporal	-0.108	0.395	0.987
Alertness warning tone	right insula	-0.076	0.550	0.987
Alertness warning tone	right isthmus cingulate	0.127	0.316	0.987
Alertness warning tone	right lateral occipital	-0.128	0.312	0.987
Alertness warning tone	right lateral orbitofrontal	-0.007	0.956	0.987
Alertness warning tone	right lingual	0.008	0.953	0.987
Alertness warning tone	right medial orbitofrontal	-0.018	0.888	0.987
Alertness warning tone	right middle temporal	-0.038	0.763	0.987
Alertness warning tone	right paracentral	0.098	0.441	0.987
Alertness warning tone	right parahippocampal	-0.028	0.828	0.987
Alertness warning tone	right pars opercularis	-0.023	0.859	0.987
Alertness warning tone	right pars orbitalis	-0.018	0.889	0.987
Alertness warning tone	right pars triangularis	0.062	0.624	0.987
Alertness warning tone	right pericalcarine	0.002	0.986	0.987
Alertness warning tone	right postcentral	-0.057	0.655	0.987
Alertness warning tone	right posterior cingulate	-0.065	0.610	0.987
Alertness warning tone	right precentral	-0.024	0.852	0.987
Alertness warning tone	right precuneus	0.032	0.801	0.987
Alertness warning tone	right rostral anterior cingulate	-0.002	0.987	0.987
Alertness warning tone	right rostral middle frontal	-0.005	0.969	0.987
Alertness warning tone	right superior frontal	0.038	0.764	0.987
Alertness warning tone	right superior parietal	0.114	0.370	0.987
Alertness warning tone	right superior temporal	0.007	0.958	0.987
Alertness warning tone	right supramarginal	-0.056	0.661	0.987
Alertness warning tone	right temporal pole	0.132	0.297	0.987
Alertness warning tone	right transverse temporal	-0.021	0.871	0.987
Phasic alertness	left banks of the superior temporal sulcus	0.205	0.105	0.667
Phasic alertness	left caudal anterior cingulate	0.089	0.486	0.703
Phasic alertness	left caudal middle frontal	0.045	0.725	0.865

Phasic alertness	left cuneus	0.151	0.235	0.667
Phasic alertness	left entorhinal	-0.233	0.064	0.667
Phasic alertness	left frontal pole	0.037	0.772	0.875
Phasic alertness	left fusiform	0.096	0.449	0.682
Phasic alertness	left inferior parietal	0.066	0.605	0.839
Phasic alertness	left inferior temporal	0.164	0.197	0.667
Phasic alertness	left insula	0.121	0.339	0.667
Phasic alertness	left isthmus cingulate	0.132	0.297	0.667
Phasic alertness	left lateral occipital	0.037	0.771	0.875
Phasic alertness	left lateral orbitofrontal	0.138	0.277	0.667
Phasic alertness	left lingual	0.060	0.636	0.859
Phasic alertness	left medial orbitofrontal	0.105	0.410	0.682
Phasic alertness	left middle temporal	0.183	0.148	0.667
Phasic alertness	left paracentral	-0.070	0.583	0.825
Phasic alertness	left parahippocampal	-0.059	0.645	0.859
Phasic alertness	left pars opercularis	0.096	0.451	0.682
Phasic alertness	left pars orbitalis	0.097	0.447	0.682
Phasic alertness	left pars triangularis	0.167	0.186	0.667
Phasic alertness	left pericalcarine	-0.126	0.321	0.667
Phasic alertness	left postcentral	-0.031	0.810	0.903
Phasic alertness	left posterior cingulate	0.198	0.116	0.667
Phasic alertness	left precentral	-0.116	0.361	0.667
Phasic alertness	left precuneus	0.007	0.953	0.968
Phasic alertness	left rostral anterior cingulate	0.116	0.363	0.667
Phasic alertness	left rostral middle frontal	0.123	0.333	0.667
Phasic alertness	left superior frontal	0.015	0.903	0.952
Phasic alertness	left superior parietal	0.004	0.974	0.974
Phasic alertness	left superior temporal	0.141	0.265	0.667
Phasic alertness	left supramarginal	0.102	0.425	0.682
Phasic alertness	left temporal pole	-0.116	0.360	0.667

Phasic alertness	left transverse temporal	0.132	0.297	0.667
Phasic alertness	right banks of the superior temporal sulcus	0.184	0.147	0.667
Phasic alertness	right caudal anterior cingulate	0.196	0.120	0.667
Phasic alertness	right caudal middle frontal	0.050	0.697	0.865
Phasic alertness	right cuneus	0.150	0.236	0.667
Phasic alertness	right entorhinal	-0.110	0.389	0.678
Phasic alertness	right frontal pole	0.162	0.200	0.667
Phasic alertness	right fusiform	0.204	0.106	0.667
Phasic alertness	right inferior parietal	0.104	0.413	0.682
Phasic alertness	right inferior temporal	0.175	0.166	0.667
Phasic alertness	right insula	0.173	0.172	0.667
Phasic alertness	right isthmus cingulate	-0.113	0.374	0.670
Phasic alertness	right lateral occipital	0.093	0.463	0.684
Phasic alertness	right lateral orbitofrontal	0.286	0.022	0.667
Phasic alertness	right lingual	0.043	0.737	0.865
Phasic alertness	right medial orbitofrontal	0.234	0.063	0.667
Phasic alertness	right middle temporal	0.156	0.217	0.667
Phasic alertness	right paracentral	-0.139	0.272	0.667
Phasic alertness	right parahippocampal	0.126	0.322	0.667
Phasic alertness	right pars opercularis	0.157	0.215	0.667
Phasic alertness	right pars orbitalis	0.138	0.275	0.667
Phasic alertness	right pars triangularis	0.197	0.118	0.667
Phasic alertness	right pericalcarine	0.049	0.702	0.865
Phasic alertness	right postcentral	-0.044	0.728	0.865
Phasic alertness	right posterior cingulate	-0.022	0.864	0.933
Phasic alertness	right precentral	-0.024	0.850	0.932
Phasic alertness	right precuneus	0.050	0.695	0.865
Phasic alertness	right rostral anterior cingulate	0.290	0.020	0.667
Phasic alertness	right rostral middle frontal	0.230	0.067	0.667

Phasic alertness	right superior frontal	0.052	0.682	0.865
Phasic alertness	right superior parietal	-0.011	0.932	0.960
Phasic alertness	right superior temporal	0.222	0.078	0.667
Phasic alertness	right supramarginal	-0.014	0.910	0.952
Phasic alertness	right temporal pole	0.185	0.144	0.667
Phasic alertness	right transverse temporal	0.257	0.040	0.667
Working memory mistakes	left banks of the superior temporal sulcus	0.088	0.489	0.962
Working memory mistakes	left caudal anterior cingulate	-0.036	0.779	0.962
Working memory mistakes	left caudal middle frontal	0.033	0.797	0.962
Working memory mistakes	left cuneus	-0.008	0.951	0.965
Working memory mistakes	left entorhinal	0.087	0.494	0.962
Working memory mistakes	left frontal pole	-0.038	0.764	0.962
Working memory mistakes	left fusiform	0.064	0.615	0.962
Working memory mistakes	left inferior parietal	0.025	0.845	0.962
Working memory mistakes	left inferior temporal	0.147	0.246	0.962
Working memory mistakes	left insula	0.139	0.275	0.962
Working memory mistakes	left isthmus cingulate	0.004	0.973	0.973
Working memory mistakes	left lateral occipital	-0.176	0.163	0.962
Working memory mistakes	left lateral orbitofrontal	0.175	0.167	0.962
Working memory mistakes	left lingual	-0.063	0.619	0.962
Working memory mistakes	left medial orbitofrontal	-0.078	0.541	0.962
Working memory mistakes	left middle temporal	0.108	0.394	0.962
Working memory mistakes	left paracentral	-0.049	0.702	0.962
Working memory mistakes	left parahippocampal	0.081	0.523	0.962
Working memory mistakes	left pars opercularis	-0.020	0.875	0.962
Working memory mistakes	left pars orbitalis	-0.061	0.632	0.962
Working memory mistakes	left pars triangularis	-0.116	0.362	0.962
Working memory mistakes	left pericalcarine	0.037	0.772	0.962
Working memory mistakes	left postcentral	-0.054	0.670	0.962
Working memory mistakes	left posterior cingulate	0.041	0.751	0.962

Working memory mistakes	left precentral	-0.050	0.692	0.962
Working memory mistakes	left precuneus	-0.027	0.829	0.962
Working memory mistakes	left rostral anterior cingulate	0.014	0.911	0.962
Working memory mistakes	left rostral middle frontal	0.125	0.324	0.962
Working memory mistakes	left superior frontal	-0.012	0.923	0.962
Working memory mistakes	left superior parietal	-0.016	0.897	0.962
Working memory mistakes	left superior temporal	-0.018	0.888	0.962
Working memory mistakes	left supramarginal	-0.012	0.927	0.962
Working memory mistakes	left temporal pole	0.101	0.426	0.962
Working memory mistakes	left transverse temporal	-0.059	0.642	0.962
Working memory mistakes	right banks of the superior temporal sulcus	-0.142	0.263	0.962
Working memory mistakes	right caudal anterior cingulate	0.023	0.858	0.962
Working memory mistakes	right caudal middle frontal	0.057	0.654	0.962
Working memory mistakes	right cuneus	0.103	0.419	0.962
Working memory mistakes	right entorhinal	0.037	0.773	0.962
Working memory mistakes	right frontal pole	0.119	0.349	0.962
Working memory mistakes	right fusiform	-0.051	0.687	0.962
Working memory mistakes	right inferior parietal	-0.030	0.811	0.962
Working memory mistakes	right inferior temporal	0.123	0.335	0.962
Working memory mistakes	right insula	-0.119	0.348	0.962
Working memory mistakes	right isthmus cingulate	0.049	0.702	0.962
Working memory mistakes	right lateral occipital	-0.052	0.686	0.962
Working memory mistakes	right lateral orbitofrontal	0.159	0.210	0.962
Working memory mistakes	right lingual	-0.058	0.651	0.962
Working memory mistakes	right medial orbitofrontal	0.074	0.559	0.962
Working memory mistakes	right middle temporal	0.066	0.606	0.962
Working memory mistakes	right paracentral	0.068	0.594	0.962
Working memory mistakes	right parahippocampal	-0.114	0.369	0.962
Working memory mistakes	right pars opercularis	-0.021	0.870	0.962

Working memory mistakes	right pars orbitalis	0.191	0.131	0.962
Working memory mistakes	right pars triangularis	-0.018	0.889	0.962
Working memory mistakes	right pericalcarine	0.143	0.259	0.962
Working memory mistakes	right postcentral	-0.092	0.468	0.962
Working memory mistakes	right posterior cingulate	0.098	0.441	0.962
Working memory mistakes	right precentral	0.081	0.525	0.962
Working memory mistakes	right precuneus	0.020	0.873	0.962
Working memory mistakes	right rostral anterior cingulate	-0.042	0.745	0.962
Working memory mistakes	right rostral middle frontal	0.055	0.669	0.962
Working memory mistakes	right superior frontal	-0.011	0.933	0.962
Working memory mistakes	right superior parietal	0.019	0.884	0.962
Working memory mistakes	right superior temporal	0.031	0.809	0.962
Working memory mistakes	right supramarginal	-0.080	0.529	0.962
Working memory mistakes	right temporal pole	0.094	0.458	0.962
Working memory mistakes	right transverse temporal	-0.018	0.889	0.962
Working memory missings	left banks of the superior temporal sulcus	0.241	0.055	0.585
Working memory missings	left caudal anterior cingulate	-0.010	0.938	0.952
Working memory missings	left caudal middle frontal	0.104	0.412	0.699
Working memory missings	left cuneus	0.215	0.088	0.607
Working memory missings	left entorhinal	0.086	0.498	0.745
Working memory missings	left frontal pole	0.148	0.242	0.607
Working memory missings	left fusiform	0.135	0.286	0.607
Working memory missings	left inferior parietal	0.164	0.195	0.607
Working memory missings	left inferior temporal	0.261	0.038	0.585
Working memory missings	left insula	0.150	0.238	0.607
Working memory missings	left isthmus cingulate	-0.053	0.675	0.856
Working memory missings	left lateral occipital	-0.146	0.249	0.607
Working memory missings	left lateral orbitofrontal	0.196	0.121	0.607
Working memory missings	left lingual	0.016	0.898	0.939
Working memory missings	left medial orbitofrontal	0.036	0.780	0.856

Working memory missings	left middle temporal	0.253	0.044	0.585
Working memory missings	left paracentral	-0.040	0.756	0.856
Working memory missings	left parahippocampal	-0.147	0.248	0.607
Working memory missings	left pars opercularis	0.048	0.708	0.856
Working memory missings	left pars orbitalis	0.085	0.504	0.745
Working memory missings	left pars triangularis	0.071	0.576	0.775
Working memory missings	left pericalcarine	0.144	0.256	0.607
Working memory missings	left postcentral	0.043	0.735	0.856
Working memory missings	left posterior cingulate	0.064	0.616	0.806
Working memory missings	left precentral	0.040	0.755	0.856
Working memory missings	left precuneus	0.221	0.079	0.607
Working memory missings	left rostral anterior cingulate	0.088	0.487	0.745
Working memory missings	left rostral middle frontal	0.190	0.132	0.607
Working memory missings	left superior frontal	0.076	0.551	0.775
Working memory missings	left superior parietal	0.236	0.060	0.585
Working memory missings	left superior temporal	0.145	0.253	0.607
Working memory missings	left supramarginal	0.185	0.143	0.607
Working memory missings	left temporal pole	-0.047	0.711	0.856
Working memory missings	left transverse temporal	0.018	0.887	0.939
Working memory missings	right banks of the superior temporal sulcus	0.041	0.745	0.856
Working memory missings	right caudal anterior cingulate	0.070	0.582	0.775
Working memory missings	right caudal middle frontal	0.108	0.395	0.699
Working memory missings	right cuneus	0.105	0.408	0.699
Working memory missings	right entorhinal	0.146	0.250	0.607
Working memory missings	right frontal pole	0.128	0.312	0.629
Working memory missings	right fusiform	0.141	0.265	0.607
Working memory missings	right inferior parietal	0.115	0.365	0.690
Working memory missings	right inferior temporal	0.239	0.057	0.585
Working memory missings	right insula	-0.017	0.895	0.939

Working memory missings	right isthmus cingulate	0.006	0.966	0.966
Working memory missings	right lateral occipital	0.147	0.247	0.607
Working memory missings	right lateral orbitofrontal	0.197	0.118	0.607
Working memory missings	right lingual	0.037	0.770	0.856
Working memory missings	right medial orbitofrontal	0.152	0.230	0.607
Working memory missings	right middle temporal	0.247	0.049	0.585
Working memory missings	right paracentral	0.040	0.755	0.856
Working memory missings	right parahippocampal	-0.139	0.274	0.607
Working memory missings	right pars opercularis	0.157	0.215	0.607
Working memory missings	right pars orbitalis	0.268	0.032	0.585
Working memory missings	right pars triangularis	0.128	0.315	0.629
Working memory missings	right pericalcarine	0.149	0.240	0.607
Working memory missings	right postcentral	0.074	0.561	0.775
Working memory missings	right posterior cingulate	0.137	0.281	0.607
Working memory missings	right precentral	0.013	0.916	0.944
Working memory missings	right precuneus	0.168	0.184	0.607
Working memory missings	right rostral anterior cingulate	0.075	0.558	0.775
Working memory missings	right rostral middle frontal	0.099	0.434	0.703
Working memory missings	right superior frontal	0.090	0.480	0.745
Working memory missings	right superior parietal	0.185	0.143	0.607
Working memory missings	right superior temporal	0.148	0.244	0.607
Working memory missings	right supramarginal	0.121	0.339	0.659
Working memory missings	right temporal pole	0.102	0.421	0.699
Working memory missings	right transverse temporal	0.112	0.379	0.697
Mental flexibility	left banks of the superior temporal sulcus	0.013	0.918	0.983
Mental flexibility	left caudal anterior cingulate	-0.084	0.511	0.856
Mental flexibility	left caudal middle frontal	0.134	0.289	0.856
Mental flexibility	left cuneus	0.102	0.421	0.856
Mental flexibility	left entorhinal	-0.204	0.106	0.856
Mental flexibility	left frontal pole	0.025	0.844	0.956

Mental flexibility	left fusiform	-0.100	0.430	0.856
Mental flexibility	left inferior parietal	0.010	0.939	0.983
Mental flexibility	left inferior temporal	0.060	0.636	0.883
Mental flexibility	left insula	-0.063	0.622	0.883
Mental flexibility	left isthmus cingulate	0.146	0.250	0.856
Mental flexibility	left lateral occipital	0.086	0.499	0.856
Mental flexibility	left lateral orbitofrontal	0.083	0.516	0.856
Mental flexibility	left lingual	0.142	0.264	0.856
Mental flexibility	left medial orbitofrontal	0.130	0.306	0.856
Mental flexibility	left middle temporal	-0.062	0.624	0.883
Mental flexibility	left paracentral	0.151	0.233	0.856
Mental flexibility	left parahippocampal	-0.110	0.388	0.856
Mental flexibility	left pars opercularis	0.051	0.689	0.919
Mental flexibility	left pars orbitalis	0.094	0.460	0.856
Mental flexibility	left pars triangularis	0.129	0.309	0.856
Mental flexibility	left pericalcarine	-0.004	0.977	0.992
Mental flexibility	left postcentral	0.117	0.358	0.856
Mental flexibility	left posterior cingulate	-0.040	0.751	0.922
Mental flexibility	left precentral	0.094	0.459	0.856
Mental flexibility	left precuneus	0.037	0.769	0.922
Mental flexibility	left rostral anterior cingulate	-0.053	0.676	0.919
Mental flexibility	left rostral middle frontal	0.149	0.239	0.856
Mental flexibility	left superior frontal	0.126	0.322	0.856
Mental flexibility	left superior parietal	0.038	0.766	0.922
Mental flexibility	left superior temporal	0.037	0.772	0.922
Mental flexibility	left supramarginal	0.001	0.992	0.992
Mental flexibility	left temporal pole	-0.105	0.407	0.856
Mental flexibility	left transverse temporal	-0.028	0.827	0.954
Mental flexibility	right banks of the superior temporal sulcus	0.007	0.955	0.984

Mental flexibility	right caudal anterior cingulate	0.073	0.568	0.866
Mental flexibility	right caudal middle frontal	0.123	0.335	0.856
Mental flexibility	right cuneus	0.080	0.532	0.856
Mental flexibility	right entorhinal	-0.098	0.441	0.856
Mental flexibility	right frontal pole	0.062	0.626	0.883
Mental flexibility	right fusiform	-0.029	0.821	0.954
Mental flexibility	right inferior parietal	0.084	0.511	0.856
Mental flexibility	right inferior temporal	0.020	0.877	0.962
Mental flexibility	right insula	0.148	0.243	0.856
Mental flexibility	right isthmus cingulate	0.110	0.385	0.856
Mental flexibility	right lateral occipital	0.037	0.773	0.922
Mental flexibility	right lateral orbitofrontal	0.182	0.150	0.856
Mental flexibility	right lingual	0.111	0.385	0.856
Mental flexibility	right medial orbitofrontal	0.010	0.940	0.983
Mental flexibility	right middle temporal	0.101	0.429	0.856
Mental flexibility	right paracentral	0.128	0.313	0.856
Mental flexibility	right parahippocampal	-0.145	0.252	0.856
Mental flexibility	right pars opercularis	0.072	0.573	0.866
Mental flexibility	right pars orbitalis	0.080	0.528	0.856
Mental flexibility	right pars triangularis	0.140	0.268	0.856
Mental flexibility	right pericalcarine	0.106	0.406	0.856
Mental flexibility	right postcentral	0.213	0.091	0.856
Mental flexibility	right posterior cingulate	0.022	0.863	0.962
Mental flexibility	right precentral	0.115	0.365	0.856
Mental flexibility	right precuneus	0.127	0.316	0.856
Mental flexibility	right rostral anterior cingulate	-0.092	0.469	0.856
Mental flexibility	right rostral middle frontal	0.189	0.135	0.856
Mental flexibility	right superior frontal	0.144	0.255	0.856
Mental flexibility	right superior parietal	0.131	0.304	0.856
Mental flexibility	right superior temporal	0.078	0.542	0.856

Mental flexibility	right supramarginal	0.113	0.373	0.856
Mental flexibility	right temporal pole	0.045	0.723	0.922
Mental flexibility	right transverse temporal	0.164	0.194	0.856
Divided attention mistakes	left banks of the superior temporal sulcus	-0.004	0.975	0.975
Divided attention mistakes	left caudal anterior cingulate	-0.019	0.883	0.975
Divided attention mistakes	left caudal middle frontal	0.056	0.662	0.975
Divided attention mistakes	left cuneus	-0.060	0.640	0.975
Divided attention mistakes	left entorhinal	0.129	0.310	0.975
Divided attention mistakes	left frontal pole	-0.074	0.560	0.975
Divided attention mistakes	left fusiform	0.117	0.359	0.975
Divided attention mistakes	left inferior parietal	-0.037	0.774	0.975
Divided attention mistakes	left inferior temporal	-0.041	0.749	0.975
Divided attention mistakes	left insula	0.035	0.782	0.975
Divided attention mistakes	left isthmus cingulate	-0.094	0.459	0.975
Divided attention mistakes	left lateral occipital	-0.087	0.494	0.975
Divided attention mistakes	left lateral orbitofrontal	0.090	0.479	0.975
Divided attention mistakes	left lingual	-0.020	0.877	0.975
Divided attention mistakes	left medial orbitofrontal	-0.317	0.011	0.555
Divided attention mistakes	left middle temporal	0.029	0.820	0.975
Divided attention mistakes	left paracentral	0.028	0.824	0.975
Divided attention mistakes	left parahippocampal	0.065	0.608	0.975
Divided attention mistakes	left pars opercularis	-0.009	0.941	0.975
Divided attention mistakes	left pars orbitalis	0.027	0.830	0.975
Divided attention mistakes	left pars triangularis	-0.042	0.743	0.975
Divided attention mistakes	left pericalcarine	0.045	0.727	0.975
Divided attention mistakes	left postcentral	0.034	0.787	0.975
Divided attention mistakes	left posterior cingulate	-0.031	0.807	0.975
Divided attention mistakes	left precentral	-0.012	0.927	0.975
Divided attention mistakes	left precuneus	-0.036	0.780	0.975
Divided attention mistakes	left rostral anterior cingulate	-0.062	0.626	0.975

Divided attention mistakes	left rostral middle frontal	0.110	0.385	0.975
Divided attention mistakes	left superior frontal	0.018	0.886	0.975
Divided attention mistakes	left superior parietal	-0.030	0.812	0.975
Divided attention mistakes	left superior temporal	0.015	0.908	0.975
Divided attention mistakes	left supramarginal	-0.064	0.617	0.975
Divided attention mistakes	left temporal pole	-0.041	0.750	0.975
Divided attention mistakes	left transverse temporal	0.113	0.376	0.975
Divided attention mistakes	right banks of the superior temporal sulcus	-0.127	0.316	0.975
Divided attention mistakes	right caudal anterior cingulate	-0.047	0.714	0.975
Divided attention mistakes	right caudal middle frontal	0.106	0.407	0.975
Divided attention mistakes	right cuneus	0.040	0.751	0.975
Divided attention mistakes	right entorhinal	-0.299	0.016	0.555
Divided attention mistakes	right frontal pole	0.187	0.138	0.975
Divided attention mistakes	right fusiform	-0.125	0.325	0.975
Divided attention mistakes	right inferior parietal	-0.119	0.347	0.975
Divided attention mistakes	right inferior temporal	-0.044	0.732	0.975
Divided attention mistakes	right insula	-0.042	0.741	0.975
Divided attention mistakes	right isthmus cingulate	0.060	0.637	0.975
Divided attention mistakes	right lateral occipital	-0.070	0.581	0.975
Divided attention mistakes	right lateral orbitofrontal	0.065	0.612	0.975
Divided attention mistakes	right lingual	-0.019	0.880	0.975
Divided attention mistakes	right medial orbitofrontal	0.054	0.670	0.975
Divided attention mistakes	right middle temporal	0.004	0.974	0.975
Divided attention mistakes	right paracentral	0.112	0.377	0.975
Divided attention mistakes	right parahippocampal	-0.029	0.818	0.975
Divided attention mistakes	right pars opercularis	-0.033	0.795	0.975
Divided attention mistakes	right pars orbitalis	0.087	0.492	0.975
Divided attention mistakes	right pars triangularis	0.138	0.278	0.975
Divided attention mistakes	right pericalcarine	0.055	0.663	0.975

Divided attention mistakes	right postcentral	-0.107	0.400	0.975
Divided attention mistakes	right posterior cingulate	-0.112	0.378	0.975
Divided attention mistakes	right precentral	0.010	0.935	0.975
Divided attention mistakes	right precuneus	-0.077	0.547	0.975
Divided attention mistakes	right rostral anterior cingulate	-0.020	0.878	0.975
Divided attention mistakes	right rostral middle frontal	0.125	0.325	0.975
Divided attention mistakes	right superior frontal	0.038	0.765	0.975
Divided attention mistakes	right superior parietal	-0.096	0.450	0.975
Divided attention mistakes	right superior temporal	0.046	0.718	0.975
Divided attention mistakes	right supramarginal	-0.140	0.271	0.975
Divided attention mistakes	right temporal pole	0.144	0.257	0.975
Divided attention mistakes	right transverse temporal	0.008	0.948	0.975
Divided attention missings	left banks of the superior temporal sulcus	-0.075	0.554	0.738
Divided attention missings	left caudal anterior cingulate	-0.271	0.030	0.512
Divided attention missings	left caudal middle frontal	-0.096	0.450	0.712
Divided attention missings	left cuneus	-0.096	0.449	0.712
Divided attention missings	left entorhinal	0.027	0.834	0.901
Divided attention missings	left frontal pole	-0.011	0.929	0.929
Divided attention missings	left fusiform	-0.138	0.276	0.695
Divided attention missings	left inferior parietal	-0.111	0.382	0.695
Divided attention missings	left inferior temporal	-0.183	0.147	0.592
Divided attention missings	left insula	-0.156	0.220	0.598
Divided attention missings	left isthmus cingulate	-0.162	0.200	0.592
Divided attention missings	left lateral occipital	-0.030	0.814	0.892
Divided attention missings	left lateral orbitofrontal	-0.020	0.875	0.919
Divided attention missings	left lingual	-0.014	0.914	0.927
Divided attention missings	left medial orbitofrontal	-0.339	0.006	0.209
Divided attention missings	left middle temporal	-0.177	0.162	0.592
Divided attention missings	left paracentral	-0.044	0.727	0.838
Divided attention missings	left parahippocampal	-0.088	0.487	0.735

Divided attention missings	left pars opercularis	-0.196	0.121	0.592
Divided attention missings	left pars orbitalis	-0.209	0.097	0.589
Divided attention missings	left pars triangularis	-0.224	0.075	0.572
Divided attention missings	left pericalcarine	0.045	0.725	0.838
Divided attention missings	left postcentral	-0.106	0.405	0.695
Divided attention missings	left posterior cingulate	-0.159	0.210	0.594
Divided attention missings	left precentral	-0.241	0.055	0.572
Divided attention missings	left precuneus	-0.116	0.363	0.695
Divided attention missings	left rostral anterior cingulate	-0.289	0.021	0.469
Divided attention missings	left rostral middle frontal	-0.065	0.608	0.760
Divided attention missings	left superior frontal	-0.165	0.193	0.592
Divided attention missings	left superior parietal	-0.113	0.375	0.695
Divided attention missings	left superior temporal	-0.168	0.185	0.592
Divided attention missings	left supramarginal	-0.147	0.248	0.649
Divided attention missings	left temporal pole	-0.078	0.538	0.735
Divided attention missings	left transverse temporal	-0.108	0.395	0.695
Divided attention missings	right banks of the superior temporal sulcus	-0.205	0.104	0.589
Divided attention missings	right caudal anterior cingulate	-0.351	0.005	0.209
Divided attention missings	right caudal middle frontal	-0.111	0.384	0.695
Divided attention missings	right cuneus	-0.064	0.615	0.760
Divided attention missings	right entorhinal	-0.078	0.539	0.735
Divided attention missings	right frontal pole	-0.020	0.878	0.919
Divided attention missings	right fusiform	-0.082	0.519	0.735
Divided attention missings	right inferior parietal	-0.119	0.350	0.695
Divided attention missings	right inferior temporal	-0.117	0.358	0.695
Divided attention missings	right insula	-0.182	0.150	0.592
Divided attention missings	right isthmus cingulate	-0.065	0.609	0.760
Divided attention missings	right lateral occipital	0.015	0.906	0.927
Divided attention missings	right lateral orbitofrontal	-0.078	0.540	0.735

Divided attention missings	right lingual	-0.105	0.409	0.695
Divided attention missings	right medial orbitofrontal	-0.224	0.076	0.572
Divided attention missings	right middle temporal	-0.058	0.648	0.787
Divided attention missings	right paracentral	-0.033	0.793	0.884
Divided attention missings	right parahippocampal	0.114	0.371	0.695
Divided attention missings	right pars opercularis	-0.163	0.197	0.592
Divided attention missings	right pars orbitalis	-0.179	0.158	0.592
Divided attention missings	right pars triangularis	-0.089	0.483	0.735
Divided attention missings	right pericalcarine	-0.037	0.774	0.877
Divided attention missings	right postcentral	-0.167	0.186	0.592
Divided attention missings	right posterior cingulate	-0.164	0.196	0.592
Divided attention missings	right precentral	-0.102	0.423	0.702
Divided attention missings	right precuneus	-0.212	0.093	0.589
Divided attention missings	right rostral anterior cingulate	-0.250	0.046	0.572
Divided attention missings	right rostral middle frontal	-0.119	0.348	0.695
Divided attention missings	right superior frontal	-0.115	0.364	0.695
Divided attention missings	right superior parietal	-0.127	0.317	0.695
Divided attention missings	right superior temporal	-0.065	0.610	0.760
Divided attention missings	right supramarginal	-0.227	0.071	0.572
Divided attention missings	right temporal pole	0.047	0.709	0.838
Divided attention missings	right transverse temporal	-0.086	0.502	0.735

Supplementary Table S9. Correlations of neuropsychological findings and cortical thickness of patients with borderline personality disorder. Abbreviations: *CFT-20 R, Culture Fair Intelligence Test; IQ, intelligence quotient; MWT-B, Multiple-choice Word Test; TIV, total intracranial volume; VLMT, Verbal Learning and Memory Test.*

Neuropsychological Test	Brain Region	r	p-value	Adjusted p-value
IQ CFT20R	Left Lateral Ventricle	-0.195	0.135	0.729
IQ CFT20R	Left Thalamus	0.066	0.618	0.824
IQ CFT20R	Left Caudate	-0.003	0.983	0.983
IQ CFT20R	Left Putamen	-0.124	0.345	0.729
IQ CFT20R	Left Pallidum	-0.136	0.302	0.729
IQ CFT20R	Left Hippocampus	0.266	0.040	0.634
IQ CFT20R	Left Amygdala	0.006	0.967	0.983
IQ CFT20R	Left Accumbens area	0.053	0.689	0.848
IQ CFT20R	Right Lateral Ventricle	-0.119	0.365	0.729
IQ CFT20R	Right Thalamus	-0.131	0.318	0.729
IQ CFT20R	Right Caudate	-0.067	0.611	0.824
IQ CFT20R	Right Putamen	-0.106	0.422	0.750
IQ CFT20R	Right Pallidum	-0.147	0.263	0.729
IQ CFT20R	Right Hippocampus	0.153	0.245	0.729
IQ CFT20R	Right Amygdala	-0.081	0.538	0.824
IQ CFT20R	Right Accumbens area	-0.034	0.796	0.909
VLMT Learning	Left Lateral Ventricle	-0.055	0.651	0.875
VLMT Learning	Left Thalamus	0.114	0.350	0.676
VLMT Learning	Left Caudate	-0.190	0.117	0.676
VLMT Learning	Left Putamen	-0.135	0.269	0.676
VLMT Learning	Left Pallidum	-0.099	0.417	0.676
VLMT Learning	Left Hippocampus	-0.111	0.365	0.676
VLMT Learning	Left Amygdala	-0.050	0.683	0.875
VLMT Learning	Left Accumbens area	-0.221	0.068	0.676
VLMT Learning	Right Lateral Ventricle	-0.040	0.747	0.875
VLMT Learning	Right Thalamus	0.029	0.811	0.875
VLMT Learning	Right Caudate	-0.162	0.182	0.676
VLMT Learning	Right Putamen	-0.125	0.308	0.676

VLMT Learning	Right Pallidum	-0.013	0.914	0.914
VLMT Learning	Right Hippocampus	-0.101	0.408	0.676
VLMT Learning	Right Amygdala	-0.098	0.422	0.676
VLMT Learning	Right Accumbens area	-0.028	0.821	0.875
VLMT Recognition	Left Lateral Ventricle	-0.199	0.100	0.803
VLMT Recognition	Left Thalamus	-0.005	0.966	0.966
VLMT Recognition	Left Caudate	0.039	0.753	0.966
VLMT Recognition	Left Putamen	0.147	0.227	0.966
VLMT Recognition	Left Pallidum	0.086	0.483	0.966
VLMT Recognition	Left Hippocampus	0.015	0.902	0.966
VLMT Recognition	Left Amygdala	-0.042	0.734	0.966
VLMT Recognition	Left Accumbens area	-0.049	0.692	0.966
VLMT Recognition	Right Lateral Ventricle	-0.279	0.020	0.322
VLMT Recognition	Right Thalamus	-0.030	0.804	0.966
VLMT Recognition	Right Caudate	0.016	0.897	0.966
VLMT Recognition	Right Putamen	0.121	0.321	0.966
VLMT Recognition	Right Pallidum	0.007	0.956	0.966
VLMT Recognition	Right Hippocampus	-0.010	0.933	0.966
VLMT Recognition	Right Amygdala	-0.118	0.333	0.966
VLMT Recognition	Right Accumbens area	-0.072	0.555	0.966
VLMT Consolidation	Left Lateral Ventricle	0.016	0.899	0.915
VLMT Consolidation	Left Thalamus	-0.223	0.065	0.825
VLMT Consolidation	Left Caudate	-0.073	0.551	0.882
VLMT Consolidation	Left Putamen	0.049	0.689	0.915
VLMT Consolidation	Left Pallidum	0.143	0.240	0.825
VLMT Consolidation	Left Hippocampus	-0.106	0.385	0.825
VLMT Consolidation	Left Amygdala	-0.013	0.915	0.915
VLMT Consolidation	Left Accumbens area	-0.126	0.302	0.825
VLMT Consolidation	Right Lateral Ventricle	-0.045	0.715	0.915
VLMT Consolidation	Right Thalamus	-0.098	0.425	0.825

VLMT Consolidation	Right Caudate	-0.034	0.781	0.915
VLMT Consolidation	Right Putamen	0.018	0.883	0.915
VLMT Consolidation	Right Pallidum	0.090	0.464	0.825
VLMT Consolidation	Right Hippocampus	-0.164	0.177	0.825
VLMT Consolidation	Right Amygdala	-0.130	0.287	0.825
VLMT Consolidation	Right Accumbens area	-0.113	0.355	0.825
Alertness no warning tone	Left Lateral Ventricle	-0.061	0.634	0.822
Alertness no warning tone	Left Thalamus	-0.013	0.916	0.916
Alertness no warning tone	Left Caudate	0.077	0.548	0.822
Alertness no warning tone	Left Putamen	0.158	0.212	0.822
Alertness no warning tone	Left Pallidum	-0.058	0.649	0.822
Alertness no warning tone	Left Hippocampus	-0.091	0.473	0.822
Alertness no warning tone	Left Amygdala	0.019	0.879	0.916
Alertness no warning tone	Left Accumbens area	0.161	0.203	0.822
Alertness no warning tone	Right Lateral Ventricle	0.096	0.449	0.822
Alertness no warning tone	Right Thalamus	-0.088	0.488	0.822
Alertness no warning tone	Right Caudate	0.090	0.478	0.822
Alertness no warning tone	Right Putamen	0.204	0.106	0.822
Alertness no warning tone	Right Pallidum	0.080	0.532	0.822
Alertness no warning tone	Right Hippocampus	-0.033	0.795	0.909
Alertness no warning tone	Right Amygdala	0.055	0.668	0.822
Alertness no warning tone	Right Accumbens area	0.139	0.273	0.822
Alertness warning tone	Left Lateral Ventricle	-0.035	0.783	0.940
Alertness warning tone	Left Thalamus	0.082	0.521	0.940
Alertness warning tone	Left Caudate	0.070	0.585	0.940
Alertness warning tone	Left Putamen	0.159	0.210	0.841
Alertness warning tone	Left Pallidum	-0.033	0.798	0.940
Alertness warning tone	Left Hippocampus	-0.025	0.843	0.940
Alertness warning tone	Left Amygdala	-0.044	0.728	0.940
Alertness warning tone	Left Accumbens area	0.162	0.201	0.841

Alertness warning tone	Right Lateral Ventricle	0.116	0.361	0.940
Alertness warning tone	Right Thalamus	-0.019	0.881	0.940
Alertness warning tone	Right Caudate	0.074	0.562	0.940
Alertness warning tone	Right Putamen	0.188	0.136	0.841
Alertness warning tone	Right Pallidum	0.040	0.751	0.940
Alertness warning tone	Right Hippocampus	-0.032	0.804	0.940
Alertness warning tone	Right Amygdala	0.001	0.991	0.991
Alertness warning tone	Right Accumbens area	0.176	0.165	0.841
Phasic alertness	Left Lateral Ventricle	-0.053	0.679	0.776
Phasic alertness	Left Thalamus	-0.165	0.191	0.624
Phasic alertness	Left Caudate	0.090	0.478	0.696
Phasic alertness	Left Putamen	0.120	0.344	0.624
Phasic alertness	Left Pallidum	0.033	0.797	0.818
Phasic alertness	Left Hippocampus	-0.162	0.200	0.624
Phasic alertness	Left Amygdala	0.125	0.325	0.624
Phasic alertness	Left Accumbens area	0.109	0.390	0.624
Phasic alertness	Right Lateral Ventricle	-0.057	0.652	0.776
Phasic alertness	Right Thalamus	-0.155	0.222	0.624
Phasic alertness	Right Caudate	0.111	0.382	0.624
Phasic alertness	Right Putamen	0.161	0.204	0.624
Phasic alertness	Right Pallidum	0.154	0.224	0.624
Phasic alertness	Right Hippocampus	-0.029	0.818	0.818
Phasic alertness	Right Amygdala	0.077	0.546	0.728
Phasic alertness	Right Accumbens area	0.111	0.384	0.624
Working memory mistakes	Left Lateral Ventricle	0.009	0.944	0.992
Working memory mistakes	Left Thalamus	0.027	0.832	0.992
Working memory mistakes	Left Caudate	-0.073	0.568	0.992
Working memory mistakes	Left Putamen	0.147	0.247	0.992
Working memory mistakes	Left Pallidum	0.096	0.449	0.992
Working memory mistakes	Left Hippocampus	0.001	0.992	0.992

Working memory mistakes	Left Amygdala	-0.098	0.440	0.992
Working memory mistakes	Left Accumbens area	-0.067	0.601	0.992
Working memory mistakes	Right Lateral Ventricle	0.085	0.505	0.992
Working memory mistakes	Right Thalamus	0.003	0.979	0.992
Working memory mistakes	Right Caudate	-0.024	0.850	0.992
Working memory mistakes	Right Putamen	0.172	0.175	0.992
Working memory mistakes	Right Pallidum	0.128	0.314	0.992
Working memory mistakes	Right Hippocampus	-0.091	0.475	0.992
Working memory mistakes	Right Amygdala	-0.061	0.631	0.992
Working memory mistakes	Right Accumbens area	0.037	0.773	0.992
Working memory missings	Left Lateral Ventricle	0.082	0.519	0.754
Working memory missings	Left Thalamus	0.003	0.981	0.981
Working memory missings	Left Caudate	0.067	0.597	0.796
Working memory missings	Left Putamen	0.164	0.194	0.702
Working memory missings	Left Pallidum	0.264	0.035	0.445
Working memory missings	Left Hippocampus	-0.126	0.321	0.702
Working memory missings	Left Amygdala	0.108	0.395	0.702
Working memory missings	Left Accumbens area	-0.046	0.717	0.864
Working memory missings	Right Lateral Ventricle	0.005	0.972	0.981
Working memory missings	Right Thalamus	0.187	0.140	0.702
Working memory missings	Right Caudate	0.117	0.358	0.702
Working memory missings	Right Putamen	0.124	0.329	0.702
Working memory missings	Right Pallidum	0.240	0.056	0.445
Working memory missings	Right Hippocampus	-0.120	0.346	0.702
Working memory missings	Right Amygdala	0.088	0.487	0.754
Working memory missings	Right Accumbens area	-0.040	0.756	0.864
Mental flexibility	Left Lateral Ventricle	0.005	0.971	0.971
Mental flexibility	Left Thalamus	0.103	0.417	0.961
Mental flexibility	Left Caudate	0.095	0.455	0.961
Mental flexibility	Left Putamen	0.082	0.520	0.961

Mental flexibility	Left Pallidum	0.109	0.393	0.961
Mental flexibility	Left Hippocampus	0.156	0.219	0.961
Mental flexibility	Left Amygdala	0.041	0.748	0.961
Mental flexibility	Left Accumbens area	-0.035	0.782	0.961
Mental flexibility	Right Lateral Ventricle	-0.066	0.605	0.961
Mental flexibility	Right Thalamus	0.151	0.234	0.961
Mental flexibility	Right Caudate	0.032	0.803	0.961
Mental flexibility	Right Putamen	0.012	0.926	0.971
Mental flexibility	Right Pallidum	0.068	0.594	0.961
Mental flexibility	Right Hippocampus	0.091	0.477	0.961
Mental flexibility	Right Amygdala	-0.026	0.836	0.961
Mental flexibility	Right Accumbens area	-0.026	0.841	0.961
Divided attention mistakes	Left Lateral Ventricle	-0.090	0.479	0.892
Divided attention mistakes	Left Thalamus	0.187	0.138	0.554
Divided attention mistakes	Left Caudate	-0.170	0.179	0.573
Divided attention mistakes	Left Putamen	-0.229	0.069	0.544
Divided attention mistakes	Left Pallidum	-0.053	0.679	0.906
Divided attention mistakes	Left Hippocampus	-0.077	0.544	0.892
Divided attention mistakes	Left Amygdala	0.002	0.988	0.999
Divided attention mistakes	Left Accumbens area	-0.081	0.526	0.892
Divided attention mistakes	Right Lateral Ventricle	-0.030	0.813	0.929
Divided attention mistakes	Right Thalamus	0.206	0.102	0.544
Divided attention mistakes	Right Caudate	-0.154	0.224	0.596
Divided attention mistakes	Right Putamen	-0.216	0.087	0.544
Divided attention mistakes	Right Pallidum	-0.075	0.557	0.892
Divided attention mistakes	Right Hippocampus	0.053	0.680	0.906
Divided attention mistakes	Right Amygdala	-0.000	0.999	0.999
Divided attention mistakes	Right Accumbens area	0.040	0.754	0.928
Divided attention missings	Left Lateral Ventricle	-0.087	0.493	0.732
Divided attention missings	Left Thalamus	0.104	0.415	0.732

Divided attention missings	Left Caudate	-0.223	0.077	0.615
Divided attention missings	Left Putamen	-0.083	0.512	0.732
Divided attention missings	Left Pallidum	0.095	0.457	0.732
Divided attention missings	Left Hippocampus	-0.139	0.274	0.732
Divided attention missings	Left Amygdala	-0.051	0.691	0.804
Divided attention missings	Left Accumbens area	-0.183	0.149	0.732
Divided attention missings	Right Lateral Ventricle	-0.049	0.703	0.804
Divided attention missings	Right Thalamus	0.128	0.314	0.732
Divided attention missings	Right Caudate	-0.230	0.067	0.615
Divided attention missings	Right Putamen	-0.139	0.273	0.732
Divided attention missings	Right Pallidum	0.076	0.549	0.732
Divided attention missings	Right Hippocampus	0.023	0.856	0.867
Divided attention missings	Right Amygdala	0.021	0.867	0.867
Divided attention missings	Right Accumbens area	-0.117	0.356	0.732

Supplementary Table S10. Correlations of neuropsychological findings and subcortical volumes of patients with borderline personality disorder. Abbreviations: *CFT-20 R, Culture Fair Intelligence Test; IQ, intelligence quotient; MWT-B, Multiple-choice Word Test; TIV, total intracranial volume; VLMT, Verbal Learning and Memory Test.*

Neuropsychological Test	Brain Region	r	p-value	Adjusted p-value
IQ CFT20R	GM/TIV corrected	0.113	0.390	0.650
IQ CFT20R	WM/TIV corrected	-0.250	0.054	0.268
IQ CFT20R	CSF/TIV corrected	0.029	0.828	0.828
IQ CFT20R	TIV corrected	0.181	0.165	0.413
IQ CFT20R	Cerebellum/TIV corrected	-0.029	0.826	0.828
VLMT Learning	GM/TIV corrected	-0.076	0.537	0.986
VLMT Learning	WM/TIV corrected	-0.062	0.610	0.986
VLMT Learning	CSF/TIV corrected	0.103	0.399	0.986
VLMT Learning	TIV corrected	0.004	0.976	0.986
VLMT Learning	Cerebellum/TIV corrected	0.002	0.986	0.986
VLMT Recognition	GM/TIV corrected	0.017	0.891	0.891
VLMT Recognition	WM/TIV corrected	0.077	0.529	0.891
VLMT Recognition	CSF/TIV corrected	-0.092	0.452	0.891
VLMT Recognition	TIV corrected	-0.029	0.813	0.891
VLMT Recognition	Cerebellum/TIV corrected	0.053	0.667	0.891
VLMT Consolidation	GM/TIV corrected	-0.104	0.394	0.657
VLMT Consolidation	WM/TIV corrected	-0.113	0.354	0.657
VLMT Consolidation	CSF/TIV corrected	0.165	0.175	0.657
VLMT Consolidation	TIV corrected	0.052	0.669	0.837
VLMT Consolidation	Cerebellum/TIV corrected	-0.002	0.987	0.987
Alertness no warning tone	GM/TIV corrected	0.026	0.838	0.838
Alertness no warning tone	WM/TIV corrected	-0.099	0.436	0.826
Alertness no warning tone	CSF/TIV corrected	0.056	0.661	0.826
Alertness no warning tone	TIV corrected	0.063	0.620	0.826
Alertness no warning tone	Cerebellum/TIV corrected	-0.328	0.008	0.041
Alertness warning tone	GM/TIV corrected	0.082	0.521	0.758
Alertness warning tone	WM/TIV corrected	-0.139	0.273	0.683
Alertness warning tone	CSF/TIV corrected	0.027	0.832	0.832

Alertness warning tone	TIV corrected	0.066	0.606	0.758
Alertness warning tone	Cerebellum/TIV corrected	-0.298	0.017	0.084
Phasic alertness	GM/TIV corrected	-0.138	0.275	0.688
Phasic alertness	WM/TIV corrected	0.057	0.654	0.817
Phasic alertness	CSF/TIV corrected	0.093	0.463	0.772
Phasic alertness	TIV corrected	0.013	0.918	0.918
Phasic alertness	Cerebellum/TIV corrected	-0.173	0.171	0.688
Working memory mistakes	GM/TIV corrected	0.018	0.890	0.988
Working memory mistakes	WM/TIV corrected	-0.002	0.988	0.988
Working memory mistakes	CSF/TIV corrected	0.052	0.682	0.988
Working memory mistakes	TIV corrected	0.117	0.357	0.988
Working memory mistakes	Cerebellum/TIV corrected	0.079	0.533	0.988
Working memory missings	GM/TIV corrected	0.027	0.830	0.830
Working memory missings	WM/TIV corrected	0.158	0.212	0.830
Working memory missings	CSF/TIV corrected	-0.075	0.553	0.830
Working memory missings	TIV corrected	0.083	0.513	0.830
Working memory missings	Cerebellum/TIV corrected	-0.044	0.731	0.830
Mental flexibility	GM/TIV corrected	-0.007	0.954	0.954
Mental flexibility	WM/TIV corrected	0.218	0.083	0.415
Mental flexibility	CSF/TIV corrected	-0.095	0.457	0.712
Mental flexibility	TIV corrected	0.076	0.553	0.712
Mental flexibility	Cerebellum/TIV corrected	0.072	0.570	0.712
Divided attention mistakes	GM/TIV corrected	0.177	0.162	0.233
Divided attention mistakes	WM/TIV corrected	0.167	0.186	0.233
Divided attention mistakes	CSF/TIV corrected	-0.236	0.060	0.233
Divided attention mistakes	TIV corrected	-0.092	0.470	0.470
Divided attention mistakes	Cerebellum/TIV corrected	0.170	0.180	0.233
Divided attention missings	GM/TIV corrected	0.066	0.602	0.602
Divided attention missings	WM/TIV corrected	0.171	0.178	0.297
Divided attention missings	CSF/TIV corrected	-0.097	0.446	0.557

Divided attention missings	TIV corrected	-0.181	0.152	0.297
Divided attention missings	Cerebellum/TIV corrected	0.175	0.166	0.297

Supplementary Table S11. Correlations of neuropsychological findings and global volume measures of patients with borderline personality disorder. Abbreviations: *CFT-20 R, Culture Fair Intelligence Test; CSF, cerebrospinal fluid; GM, grey matter; IQ, intelligence quotient; MWT-B, Multiple-choice Word Test; TIV, total intracranial volume; VLMT, Verbal Learning and Memory Test; WM, white matter.*
